# Maintenance of Self-Renewal and Pluripotency in J1 Mouse Embryonic Stem Cells through Regulating Transcription Factor and MicroRNA Expression Induced by PD0325901

**DOI:** 10.1155/2016/1792573

**Published:** 2015-12-07

**Authors:** Zhiying Ai, Jingjing Shao, Xinglong Shi, Mengying Yu, Yongyan Wu, Juan Du, Yong Zhang, Zekun Guo

**Affiliations:** ^1^College of Life Sciences, Northwest A&F University, Yangling, Shaanxi 712100, China; ^2^Key Laboratory of Animal Biotechnology, Ministry of Agriculture, Northwest A&F University, Yangling, Shaanxi 712100, China; ^3^College of Veterinary Medicine, Northwest A&F University, Yangling, Shaanxi 712100, China

## Abstract

Embryonic stem cells (ESCs) have the ability to grow indefinitely and retain their pluripotency in culture, and this self-renewal capacity is governed by several crucial molecular pathways controlled by specific regulatory genes and epigenetic modifications. It is reported that multiple epigenetic regulators, such as miRNA and pluripotency factors, can be tightly integrated into molecular pathways and cooperate to maintain self-renewal of ESCs. However, mouse ESCs in serum-containing medium seem to be heterogeneous due to the self-activating differentiation signal of MEK/ERK. Thus, to seek for the crucial miRNA and key regulatory genes that establish ESC properties in MEK/ERK pathway, we performed microarray analysis and small RNA deep-sequencing of J1 mESCs treated with or without PD0325901 (PD), a well-known inhibitor of MEK/ERK signal pathway, followed by verification of western blot analysis and quantitative real-time PCR verification; we found that PD regulated the transcript expressions related to self-renewal and differentiation and antagonized the action of retinoic acid- (RA-) induced differentiation. Moreover, PD can significantly modulate the expressions of multiple miRNAs that have crucial functions in ESC development. Overall, our results demonstrate that PD could enhance ESC self-renewal capacity both by key regulatory genes and ES cell-specific miRNA, which in turn influences ESC self-renewal and cellular differentiation.

## 1. Introduction

Embryonic stem cells (ESCs) derived from the inner cell mass of mammalian embryos have the unique ability to grow indefinitely in culture while retaining their pluripotency [1]. This self-renewal capacity is established through the integration of several molecular pathways controlled by key regulatory genes and complex epigenetic modifications. Oct4, Sox2, and Nanog [2, 3], recognized as fundamental regulatory genes, cooperate with additional core transcriptional regulators such as Stat3, Esrrb, Klf4, Myc, and Sall4 to maintain mouse ESC properties [3]. DNA methylation, as one of the key mechanisms of epigenetic regulations, is important to the establishment of pluripotency in ESCs [4]. Moreover, functional studies have shown that inhibition of de novo DNA methyltransferase by PRDM14 was able to block ESC from naive inner cell mass- (ICM-) like state to a primed epiblast-like state [5, 6]. Meanwhile, microRNAs (miRNAs), as an important mechanism of epigenetic regulation, play crucial roles in normal ESC self-renewal and cellular differentiation by tightly controlling ESC self-renewal and differentiation pathways [7, 8]. These multiple epigenetic regulators and pluripotency factors can be tightly integrated into one or several molecular pathways and cooperate to maintain self-renewal of ESCs [9, 10].

Mouse ESCs (mESCs) can be maintained in serum-containing medium with the presence of leukemia inhibitor factor (LIF) or serum-free N2B27 medium supplemented with two small molecule inhibitors (2i) of CHIR99021 (CHIR) and PD0325901 [11, 12]. It has been discovered that several molecular pathways including JAK/STAT, BMP/SMAD, Wnt/*β*-catenin, and MEK/ERK are the underlying basis of these two ESC media for supporting mESC pluripotency in culture. However, mESCs in serum-containing medium are heterogeneous, which is different from a homogeneous state of ESC in serum-free N2B27 medium supplemented with 2i. This is due to the self-activating differentiation signal of MEK/ERK that triggers differentiation of ESCs, which might result in the heterogeneous state of ESCs. Recent studies have identified that PD is one of the inhibitors of MEK/ERK pathway stimulated by fibroblast growth factor-4 (Fgf4) in mESCs [11, 13]. Inactivation of MEK/ERK by PD restricts the differentiation of ESCs [14], and this effect is majorly mediated by enhancing Nanog expression [15–17]. However, very little is known about the other possible mechanisms that function in this process. For example, the role of miRNAs has not been investigated so far when MEK/ERK signaling cascade was blocked. Clarification of miRNAs functions in MEK/ERK signaling will provide further insight into mechanisms that ESCs maintain their intrinsic properties.

The miRNAs are small noncoding RNAs that regulate mRNA stability and/or translational efficiency [18]. Most miRNA genes are transcribed from either miRNA genes or intronic sequences of protein coding genes by RNA polymerase II to generate a stem-loop containing primary miRNA (pri-miRNA) [19]. The hairpin embedded in pri-miRNA is recognized by the RNA-binding protein Dgcr8, which directs the RNase III enzyme Drosha to cleave the base of the hairpin [20, 21]. Following cleavage by the Drosha-Dgcr8 complex, the released short hairpin called precursor miRNA (pre-miRNA) is then transported by the Exportin-5/Ran-GTP complex to the cytoplasm, where Dicer, together with Trbp2, cleaves it into a single short 18–25 nt dsRNA [22]. Each of them can be recruited into RNA-induced silencing complex (RISC). This complex targets mRNAs via base pairing between the miRNA and mRNA, resulting in the regulation of various aspects of stem cell functions including the maintenance and induction of pluripotency for reprogramming [7, 23]. Several lines of evidence further indicated the global function of miRNAs in Dicer or DGCR8-deficient mESCs [24–27]. Additionally, individual miRNA function has also been revealed in ESCs [28–30]. Thus, in order to dissect how epigenetic regulator including miRNA and key regulatory genes establish J1 mouse ESC properties in a defined molecular pathway, we identified MEK/ERK signal-related miRNAs and genome-wide regulation profiles of J1 mESCs stimulated by PD using small RNA deep-sequencing and microarray analysis followed by subsequent verification. We demonstrated that PD enhances ESC self-renewal capacity by not only key regulatory genes but also ESC-specific miRNA, which in turn mediates ESC self-renewal and cellular differentiation.

## 2. Materials and Methods

### 2.1. ESC Culture

The mouse J1 ESC line purchased from the American Type Culture Collection (Manassas, VA, USA) was cultured in 0.1% (w/v) gelatin coated tissue culture plates without feeders in ESC media [knockout Dulbecco's modified Eagle's medium supplemented with 15% (v/v) knockout serum replacement, 0.1 mM *β*-mercaptoethanol, 1x nonessential amino acids, 2 mM GlutaMax, 50 U/mL penicillin, 50 *μ*g/mL streptomycin (Life Technologies Inc., Grand Island, NY, USA), and 1000 U/mL LIF (ESGRO, Millipore, USA)]. 293T cell line was cultured at 37°C humidified air with 5% CO2 in Dulbecco's modified Eagle medium supplemented with 10% fetal bovine serum.

### 2.2. Reagents and Antibodies

PD0325901, DMSO, and mouse anti-GAPDH were purchased from Sigma-Aldrich. The primary antibodies used were rabbit anti-Nanog (CST, Danvers, MA, USA), rabbit anti-Klf4 (Boster, Wuhan, China), mouse-anti-c-Myc (Santa Cruz, CA, USA), goat anti-Tet1 (Santa Cruz), rabbit anti-5hmC (Active Motif, Carlsbad, CA, USA), rabbit anti-Ezh2 (Abcam, Cambridge, UK), rabbit anti-H3K27me3 (Abcam), mouse anti-Oct3/4 (Santa Cruz), and mouse anti-Sox2 (Santa Cruz). Alexa Fluor 555-labeled goat anti-rabbit/mouse IgG and anti-rabbit/mouse horseradish peroxidase-conjugated secondary antibody were obtained from the Beyotime Institute of Biotechnology (Nantong, Jiangsu, China).

### 2.3. RT-qPCR

The total RNA was isolated from cultured cells using the Trizol reagent (Life Technologies). First-strand cDNA synthesis was performed using the SYBR PrimeScript RT reagent Kit (Perfect Real Time) (Takara, Dalian, China) according to the manufacturer's instructions. qPCR was performed using SYBR Premix Ex Taq II (Takara). RT-qPCR was performed in an ABI StepOne Plus PCR System (Applied Biosystems, California, USA) with SYBR Premix Ex TaqTM (Takara). The forward and reverse primers used for real-time PCR were shown in Supplementary Table  4 in Supplementary Material available online at http://dx.doi.org/10.1155/2016/1792573. The expression of each gene was defined from the threshold cycle (Ct), and relative expression levels were calculated by using the 2^−ΔΔCT^ method after normalization with reference to expression of the housekeeping gene Gapdh. The gene expression ratio was shown as mean ± SD from three independent experiments.

### 2.4. Western Blot Analysis

Cultured cells were lysed in RIPA buffer. Equal amounts of proteins were separated by 10% polyacrylamide gels and transferred to PVDF membranes (Millipore, MA, USA) for 2 h at 100 V. After blocking nonspecific binding by soaking the filters in 5% skim milk, the desired proteins were immunodetected with the respective antibodies that followed autography using SuperSignal West Pico substrate (Thermo Scientific, IL, USA) according to the manufacturer's instructions.

### 2.5. Immunofluorescence Staining

Cells were fixed in 4% paraformaldehyde for 20 min and incubated at 37°C in blocking buffer (PBS containing 5% BSA and 0.2% Triton X-100). Cells were incubated in the presence of primary antibodies at 4°C overnight and then washed three times in PBS. Cells were then incubated with Alexa Fluor 555 secondary antibody for 1 h at 37°C. Nuclei were stained with DAPI. Immunofluorescence staining was visualized and imaged by a confocal microscope (Nikon, Tokyo, Japan).

### 2.6. Microarray-Based Gene Expression Profiling and Small RNA Deep-Sequencing

ESCs were cultured on gelatin coated 6-well plates, then PD was added to medium at a final concentration of 1 *μ*M, and an equal volume of DMSO was added to medium for control cells. For each treatment three independent experiments were conducted to prepare the samples. At 24 h after treatment, total RNA was extracted using Trizol reagent (Life Technologies) following the manufacturer's instructions. RNA integrity was checked by an Agilent Bioanalyzer 2100 system (Agilent Technologies, Santa Clara, CA, USA). Qualified total RNA of each sample was divided into two copies, one for microarray experiment and the other for small RNA deep-sequencing. The microarray experiment was performed as described previously [31, 32].

For small RNA sequencing, the total RNA from three independent experiments of each treatment was pooled, respectively. Small RNA library construction and sequencing were performed by Beijing Genomics Institute (Shenzhen, China). Briefly, sRNA (18 to 30 nt) was gel purified and ligated to the 39 and 59 adaptor. The ligated products were reverse-transcribed, followed by acrylamide gel purification and PCR amplification to generate sRNA libraries. The library was loaded on an Agilent 2100 Bioanalyzer system to check size, purity, and concentration. Libraries were sequenced on an IlluminaHiSeq 2000 sequencing system (Illumina, San Diego, CA, USA). Sequencing data has been submitted to the Gene Expression Omnibus (GEO) (accession ID: GSE67570).

### 2.7. Gene Ontology (GO) and KEGG Pathway Analysis

Data screening was carried out based on a gene expression fold change of >1.5 and statistical significance of *p* < 0.05. Biological themes of the differentially expressed genes were identified by the biological processes of GO categories using the online tool of the Database for Annotation, Visualization, and Integrated Discovery (DAVID) [33]. KEGG pathway analysis was performed using the SAS online program (http://sas.ebioservice.com/portal/root/molnet_shbh/index.jsp) with the thresholds of count > 10.

### 2.8. Dual-Luciferase Reporter Assay

Pathway reporter vectors pAP1-TA-luc, pAP1 (PMA)-TA-luc, pISRE-TA-luc, pP53-TA-luc, and the negative control pTA-luc were purchased from Clontech Laboratories, Inc. (Mountain View, CA, USA). Other signaling transduction reporter vectors including pCRE-TA-luc and pGRE-TA-luc were constructed in our laboratory by inserting their cis-acting DNA binding sequence into the multiple cloning sites of pTA-luc [31]. Luciferase assays were performed with the dual-luciferase reporter assay system (Promega) according to the manufacturer's instructions. Briefly, pathway reporter vectors and pRL-SV40 were cotransfected into ESCs by Lipofectamine 2000 (Invitrogen) according to the manufacturer's protocol. At 24 h after transfection, 1 *μ*M PD or an equal volume of DMSO was added to culture medium for another 24 h. Cells were then lysed in passive lysis buffer and luciferase activity was measured on a VICTOR X5 Multilabel Plate Reader (PerkinElmer, Norwalk, CT, USA).

### 2.9. MicroRNA and qPCR Analysis

The miRNAs expression was validated by poly(A)-tailed qPCR. Total RNA was extracted from PD-treated or control sample using Trizol reagent, and 2 *μ*g of RNA was reverse-transcribed to cDNA using miScript II RT Kit (Qiagen GmbH, Hilden, Germany) according to the manufacturer's instructions. qPCR was performed using SYBR Premix Ex Taq II (Takara) on a StepOne Plus PCR System (Applied Biosystems). All reactions were performed at 95°C for 15 min to activate the HotStarTaq DNA Polymerase. This process was followed by 40 cycles of 95°C for 5 s and 60°C for 30 s. The specificity of the primer application was examined by the analysis of a melting curve. The relative expression of miRNA was normalized to small nuclear RNA (Rnu6) expression and relative to the control. Data were expressed as the fold change = 2^−ΔΔCT^.

### 2.10. Plasmid Constructs

The coding sequence (CDS) of Nanog that contain putative miRNA binding site was amplified from J1 ESC cDNA by PCR. The PCR primers were as follows: forward primer, 5′-CCGCTCGAGATGAGTGTGGGTCTTCCTGGTCC-3′ (underlined letters indicate XhoI restriction site), and reverse primer, 5′-ATAAGAATGCGGCCGCTCATATTTCACCTGGTGGAG  TCACAG-3′ (underlined letters indicate NotI restriction site). It was then cloned into the psiCHECK-2 vector (Promega), yielding psiCHECK-2-*Nanog*. The miR-296-5p mimics were purchased from Shanghai GenePharma (Shanghai, China). For mimics interference experiments, J1 ESCs were transfected with the indicated mimics (50 nM final concentrations) for 24 h using Lipofectamine 2000 (Invitrogen).

### 2.11. Statistical Analysis

Numerical data were presented as mean ± standard deviation (SD), and statistical significance was analyzed with a two-tailed Student's *t*-test. A value of *p* < 0.05 was considered significant.

## 3. Results

### 3.1. Suppression of MEK/ERK Signaling Promotes Self-Renewal and Colony Morphology of mESCs

**Figure 1:**
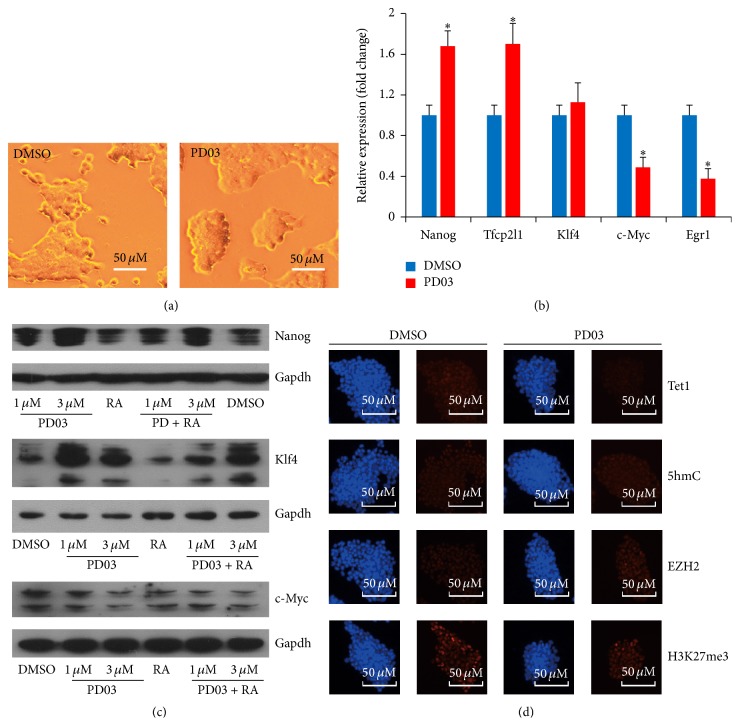
Suppression of MEK/ERK signaling promotes self-renewal and colony morphology of mESCs. (a) PD promotes colony morphology of mESCs. J1 mESCs were treated with 1 *μ*M PD or equal volume of DMSO for 24 h. Morphological changes were observed and recorded under a phase contrast microscope. Scale bar = 50 *μ*m. (b) PD influences the expression pluripotent factors. ESCs were treated with or without 1 *μ*M PD for 24 h; then the expression levels of* Nanog*,* Tfcp2l1*,* Klf4*, c-*Myc*, and* Egr1* were analyzed by RT-qPCR. Gapdh was used as a normalization control. Error bars indicate mean ± SD of three independent experiments, ^*∗*^ *p* < 0.05 compared with controls. (c) PD antagonizes RA-induced differentiation of ESCs. ESCs were treated with the indicated concentration of PD and/or together with 1 *μ*M RA for 24 h; equal volume of DMSO was added for control samples. Then the protein expression levels of Nanog, Klf4, and c-Myc were analyzed by western blot. Gapdh was used as a normalization control. (d) PD do not influence epigenetic regulation of ESCs. ESCs were treated with the 1 *μ*M PD or equal volume of DMSO for 24 h. Immunofluorescence staining assay was used for analysis of the expression level of 5hmC, Tet1, Ezh2, and H3K27me3. Nuclei were stained with DAPI; scale bar = 50 *μ*m.

Mouse ESCs are derived and maintained by using a combination of the cytokine LIF to activate STAT3 and either serum or bone morphogenetic protein (BMP) to induce inhibitor of differentiation proteins [34]. However in these processes, their differentiation involves autoinductive stimulation of the MEK/ERK pathway by Fgf4 [13, 14]. To determine the exact contribution of the suppression of MEK/ERK signaling to the undifferentiated states of mESCs, J1 mESCs cultured in gelatin coated dishes with LIF (1000 U/mL) were treated with 1 *μ*M PD for 24 h. In the presence of LIF, PD significantly promoted the formation of typical J1 mouse ESC morphology as cultured on feeder-free plates, which was smooth and tightly protuberant when PD was added (Figure 1(a)). However, after being cultured under the feeder-free condition for 3–5 passages, most J1 mESCs colonies lost typical morphology (Figure S1A, right). We then detected pluripotency of J1 mESCs cultured in these conditions for 3 passages by alkaline phosphatase (AP) activity and western blot assays and found that J1 mESCs showed AP activity in contrast to 3T3 cells, which were used for negative control (Figure S1A) and expressed high levels of Nanog and Oct4 (Figure S1B). Thus, J1 mESCs were pluripotent in these conditions when adding PD. Furthermore, the addition of PD and the expression levels of pluripotent factors* Tfcp2l1* and* Nanog* were promoted as measured by quantitative real-time PCR (RT-qPCR) (Figure 1(b)).* Egr1*, a target of the MEK/ERK signaling pathway, was repressed by MEK inhibitor PD (Figure 1(b)). Next, we treated J1 mESCs with PD or equal volume of DMSO for 24 h and then assessed the protein induction of pluripotent factors by PD. Western blot showed that Nanog and Klf4 protein expression levels were upregulated in contrast to control sample (Figure 1(c)); another small molecule SC1, a well-known inhibitor of MEK/ERK signal pathway, also confirmed these results (Figure S2A). However, Myc was repressed significantly (Figure 1(c)). Previous studies demonstrate that mESCs treated with 1 *μ*M retinoic acid (RA) can be induced to differentiate. As indicated in Figure 1(c), Nanog, Klf4, and Myc were significantly repressed by RA. However the expression levels of these two pluripotent factors were able to be rescued by the addition of 1 *μ*M or 3 *μ*M PD, respectively. We also confirmed these results by immunostaining and RT-qPCR (Figure S3). These results indicate that PD is positive for the maintenance of the undifferentiated state of ESCs. PD could promote self-renewal of mESCs by inducing the expression of pluripotency genes. Moreover, PD could antagonize RA-induced differentiation of ESCs.

To investigate alterations of global epigenetic modifications that were involved in DNA methylation in PD-treated ESCs, we performed immunofluorescence staining to examine epigenetic changes (Figure 1(d)). Previous studies indicate that 5-hydroxymethyl cytosine (5hmC) exists at high levels in mESCs, and its level significantly decreases after mESC differentiation [35]. However, the 5hmC modification level in J1 ESCs was unchanged, although a slight reduction of Tet1 was caused after PD treatment (Figure 1(d), upper panel). Moreover, the global histone H3 lysine 27 trimethylation (H3K27me3) modification level and Ezh2 expression level were also unchanged after PD treatment (Figure 1(d), lower panel).

### 3.2. Transcripts Involved in Self-Renewal and Differentiation Were Regulated by PD

**Figure 2:**
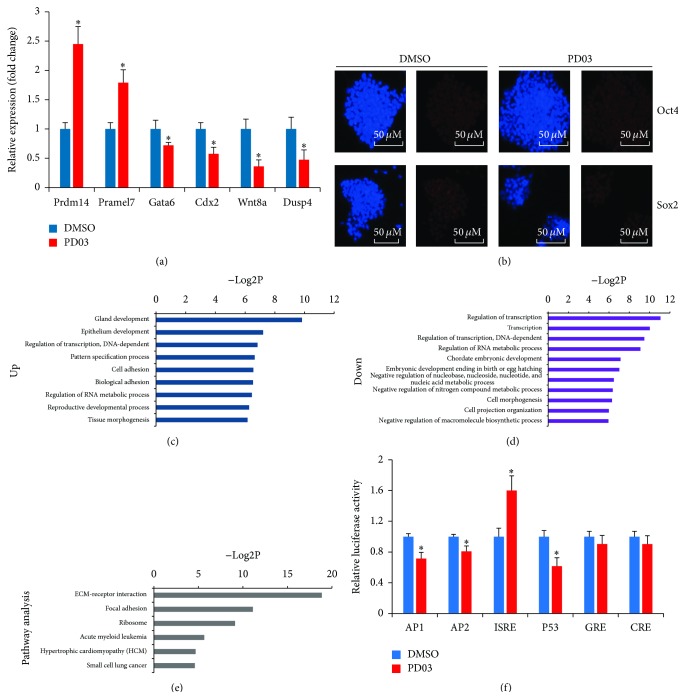
Transcripts involved in self-renewal and differentiation were regulated by PD. (a) qPCR validation of the microarray data. Cells were treated with 1 *μ*M PD or equal volume of DMSO for 24 h. The expression levels of Prdm14, pramle7, Gata6, Cdx2, Wnt8a, and Dusp4 were detected by RT-qPCR. Error bars indicate mean ± SD of three independent experiments, ^*∗*^ *p* < 0.05 compared with controls. (b) The expression of Oct4 and Sox2. Cells were treated with 1 *μ*M PD or equal volume of DMSO for 24 h. Immunofluorescence staining assay was used for analysis of the expression level of Oct4 and Sox2. Nuclei were stained with DAPI; scale bar = 50 *μ*m. (c and d) GO annotation of PD-regulated genes. GO term enrichment of the “biological process” category of PD-regulated genes. GO terms ranked according to the −Log2P of upregulated genes (count > 10) (c) or downregulated genes (d) were plotted. (e) KEGG pathway analysis of differentially expressed genes. KEGG pathway analysis of differentially expressed genes in PD-treated J1 mESCs. This result was ranked according to the −Log2P of PD-regulated genes (count > 10). (f) Dual-luciferase reporter assay to identify signaling transduction pathways regulated by PD. Pathway reporter vectors (including negative control) and internal control pRL-SV40 were cotransfected by Lipofectamine 2000. 24 h after transfection, 1 *μ*M PD or an equal volume of DMSO was added to cell medium for another 24 h. Luciferase activity is presented relative to negative control pTA-luc. Data are presented as mean ± SD of three independent experiments, ^*∗*^ *p* < 0.05.

To investigate how PD affects the ES cell fate, we performed genome-wide expression microarray analysis of J1 ES cells cultured with or without PD for 24 h (GEO ID number: GSE67534). Messenger RNAs with fold changes greater than 1.5 and *p* values less than 0.05 were presented in Supplementary Table  1. A total of 1206 differentially expressed genes were identified in PD-treated J1 mESCs compared with control-treated cells, of which 763 genes were upregulated and 443 were downregulated. From Table S1, we found that beside the well-known pluripotency-associated genes identified above (Nanog, Tfcp2l1; Figure 1(b)), other pluripotency-related genes such as Pramel7 and Prdm14 were also upregulated in J1 ES cells after 1 *μ*M PD treatment. Ectopic expression of Pramel7 inhibits differentiation and enhances ESC self-renewal, while Pramel7 knockdown induces differentiation and depresses lineage-specific markers [36, 37]. Prdm14 ensures naive pluripotency by recruiting PRC2 [5, 38]. On the other hand, genes associated with development or tissue formation, such as Gata6, Cdx2, Wnt8a, and Dusp4, were significantly downregulated. Cooperating with Brachyury, Cdx2 is reported to induce ESCs to form mesoderm through BMP-induced differentiation [39]. The RT-qPCR was performed for the part of the indicated genes to confirm the objective reliability of the gene expression changes (Figure 2(a)), and consistent results were obtained.

Based on expression profiling, Oct4, Sox2, and Klf4 had no significant expression changes, while Myc (c-Myc) transcript was downregulated, which were further verified by qPCR examination (Figure 1(b); Oct4 and Sox2, data not shown). We then reevaluated the expression of Oct4 and Sox2 using immunofluorescence assay and found that Oct4 and Sox2 were not affected by 1 *μ*M PD treatment for 24 h (Figure 2(b)). Although Klf4 mRNA did not respond to PD signal (Figure 1(b)), Klf4 protein expression level was promoted by PD (Figure 1(c)). Myc gene examinations showed the consistent results (Figures 1(b) and 1(c)). Thus, these results demonstrate that suppression of ERK1/2 signaling pathway by 1 *μ*M PD can promote expressions of key pluripotency-related gene including Nanog, Klf4, and Tfcp2l1 and suppress expressions of differentiation-inducing genes. These findings also indicate that PD contributes to the undifferentiated state of ESCs.

Functional annotation of differentially expressed genes by Gene Ontology (GO) revealed that PD-upregulated genes were significantly enriched for terms linked to developmental processes, cell adhesion, regulation of transcription, and morphogenesis (Figure 2(c)). PD-downregulated genes were highly enriched for terms associated with developmental processes, metabolic processes, transcriptional regulation, and biosynthetic processes (Figure 2(d)). Kyoto Encyclopedia of Genes and Genomes (KEGG) pathway analysis showed that PD-regulated genes are involved in the ECM-receptor interaction, the focal adhesion, and metabolic processes (Figure 2(e)). To investigate the observed effects of PD on mESCs that were not solely the result of MEK/ERK signaling, we performed luciferase reporter assays using signal transduction reporter plasmids [31, 32]. J1 mESCs were transfected with reporter plasmids that represented the signal transduction pathways of JAK-STAT (pISRE-TA-luc), JNK/p38 and PKA (pAP1-TA-luc and pCRE-TA-luc), PKC/MAPK (pAP2-TA-luc), Glucocorticoid/HSP90 (pGRE-TA-luc), and p53 (pP53-TA-luc). 24 h after transfection, 1 *μ*M PD or an equal volume of DMSO was added to cell medium for another 24 h. As shown in Figure 2(f), PD treatment was able to decrease the luciferase activity of JNK/p38, PKC/MAPK, and p53 significantly, confirming that PD inhibits these three signaling pathways in J1 mESCs; another small molecule SC1 also confirmed these results (Figure S2B). However PD was able to increase the luciferase activity of JAK-STAT, indicating that PD promotes JAK-STAT signaling pathway. Collectively, PD treatment alters the expression of transcription factors in J1 mESCs and fine-tunes the signaling pathways to maintain the characteristics of stem cells.

### 3.3. Small RNA Deep-Sequencing of PD-Treated mESCs

**Figure 3:**
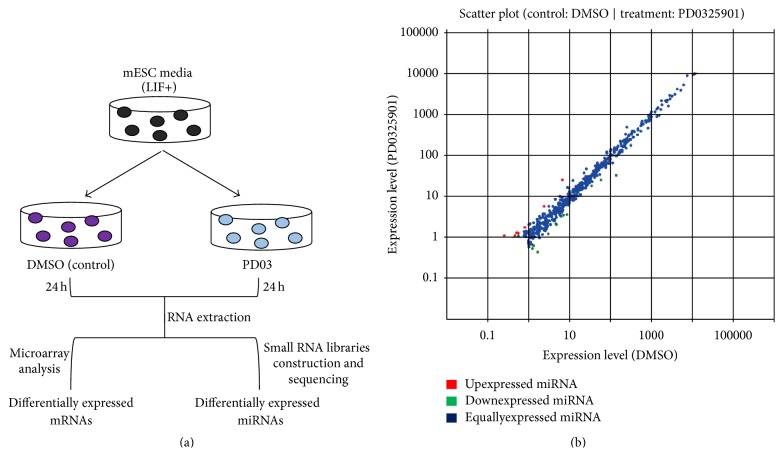
Experimental scheme for sample preparation of small RNA deep-sequencing and microarray analysis. (a) Experimental scheme for small RNA deep-sequencing and microarray analysis. J1 mESCs cultured in LIF containing media were treated with 1 *μ*M PD or equal volume of DMSO (control) for 24 h, and then the total RNAs were extracted and qualified RNAs were analyzed by microarray gene expression profiling and small RNA deep-sequencing to identify differentially expressed mRNAs and miRNAs. (b) Comparison of the known miRNA expression between DMSO- and PD-treated samples. The scatter plots show the distribution of the detected miRNAs with or without 1 *μ*M PD treatment in mESC medium for 24 h. Significant regulated miRNAs with 1.5-fold change are marked red (upregulated) and green (downregulated).

Although key regulatory genes have been well disclosed in ERK1/2 signaling cascade pathway in mESCs, ERK1/2-related miRNAs have not been investigated so far. To identify the ERK1/2 signal-related miRNAs in ESCs, we performed small RNA sequencing using small RNA deep-sequencing technology in J1 mESCs treated with 1 *μ*M PD or equal volume of DMSO for 24 h (Figure 3(a)). Totally, 18,870,345 clean reads for control-treated cells (control) and 20,944,808 clean reads for the PD-treated sample (PD) were, respectively, extracted after removal of low-quality sequences, and the 5′ and 3′ adapters, pollution reads, and reads smaller than 18 nucleotides. Scatter plots showed the general trend of miRNA expression changes after PD stimulation (Figure 3(b)). After mapping the clean reads against the GenBank noncoding RNA database and the Rfam database, we found noncoding RNAs (ncRNAs), such as rRNA, scRNA, tRNA, snRNA, snoRNA, and other ncRNAs. Then, small RNA reads were mapped against introns and exons of mRNAs to find and excise the degraded fragments of mRNA in the small RNA tags. Finally, the clean reads were aligned to miRBase (Release 18) allowing only perfect matches.

**Figure 4:**
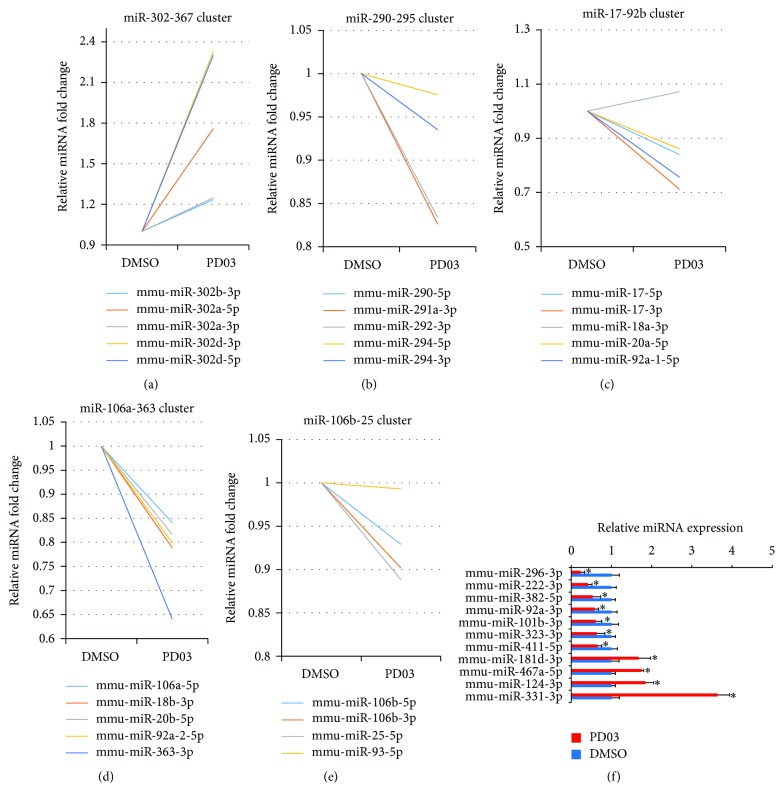
PD regulate the expression of the ESCC family of miRNAs in mESCs. (a–e) Relative fold change of mature ESCC family of miRNAs. J1 mESCs were treated with 1 *μ*M PD or equal volume of DMSO (control) for 24 h, and then the total RNAs were extracted and qualified RNAs were analyzed by small RNA deep-sequencing to identify differentially expressed miRNA. miR-302-367 cluster, miR-290-295 cluster, miR-17-92b cluster, miR-106a-363 cluster, and miR-106b-25 cluster in control and PD-treated J1 mESCs detected by small RNA deep-sequencing. (f) RT-qPCR validation of differentially expressed miRNA in PD-treated J1 mESCs. J1 mESCs were treated with 1 *μ*M PD for 24 h, and then the expression of miRNAs was determined by RT-qPCR. Error bars indicate mean ± SD of three independent experiments, ^*∗*^ *p* < 0.05 compared with controls.

After performing fold change analysis, we identified 89 differentially expressed miRNAs in J1 mESCs treated with PD compared with the control library, in which 26 miRNAs were upregulated and 63 miRNAs were downregulated by 1.5-fold or greater (Table S2). We noted that ~70% of miRNAs (63 out of 89) in the PD-treated samples were downregulated, and many miRNAs have been studied in pluripotent cells. The miR-302-367, miR-290-295, miR-17-92b, miR-106a-363, and miR-106b-25 cluster of miRNAs belong to the ESC-specific cell cycle (ESCC) family of miRNAs. The miR-302-367 cluster is expressed specifically in pluripotent ESCs, and its overexpression promotes iPS cell generation efficiency in mouse fibroblasts using three exogenous factors (Oct4, Klf4, and Sox2). The miR-290-295 cluster promotes pluripotency maintenance via regulating cell cycle phase distribution. Our sequencing data showed that the expression of miR-302a and miR-302d was upregulated by 1 *μ*M PD (Figure 4(a)), but the other ESCC miRNAs were downregulated following PD treatment (Figures 4(b)–4(e)). The differential expression levels of several miRNAs were confirmed by quantitative real-time PCR (RT-qPCR) (Figure 4(f)).

### 3.4. ERK1/2 Signal-Related miRNAs Regulate Nanog Expression and Promote Homogeneous ESC

**Figure 5:**
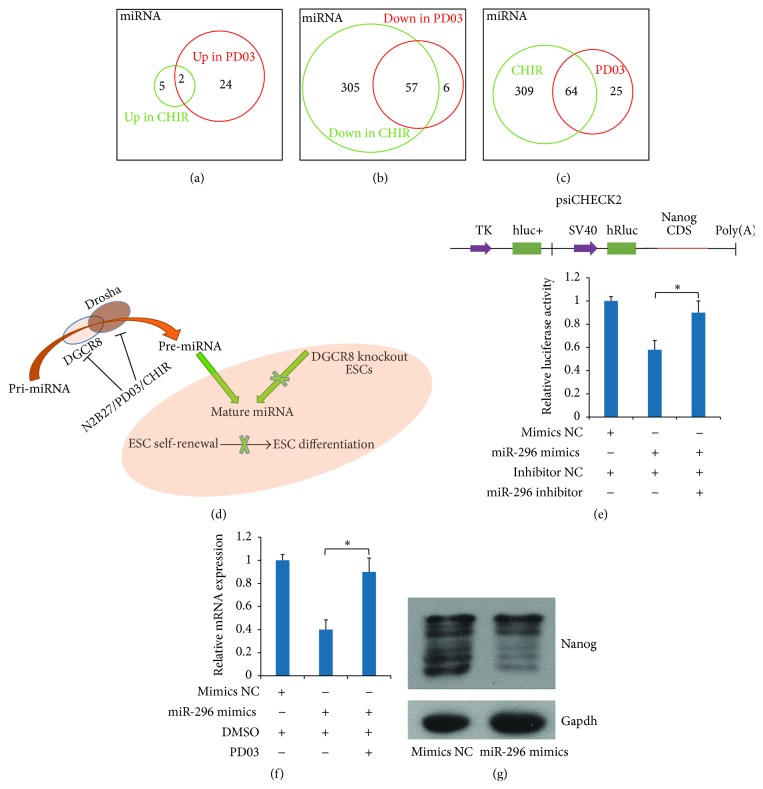
MEK/ERK signal-related miRNAs promote homogeneous ESC. (a–c) Venn diagram shows the differential expression of miRNA in PD- and CHIR-treated ESCs. Venn diagram showed the upregulated miRNAs (a) and the downregulated miRNAs (b) in PD- and CHIR-treated ESCs, respectively. The global differential miRNAs between CHIR- and PD-treated ESCs are shown in (c). (d) Schematic diagram of the miRNA biosynthesis and functions in maintaining the undifferentiated state of mESCs. PD and CHIR influence Dgcr8-Drosha complex activity. → means active and ⊥ means inactive. (e) miR-296 mimics regulated Nanog expression in a posttranscriptional regulation manner. Schematic representation of the 3′-UTR reporter constructs in the upper panel. TK, hluc+, SV40, and hRluc represent HSV-TK promoter, firefly luciferase gene, SV40 early enhancer/promoter, and Renilla luciferase gene, respectively. In the lower panel, psiCHECK2-Nanog-CDS or psiCHECK2 control plasmid was cotransfected with mimics NC or miR-296 mimics/inhibitor into 293T cells. At 24 h after incubation, 1 *μ*M PD or an equal volume of DMSO was added to cell medium for another 24 h. Luciferase activity is presented relative to negative control pTA-luc. Data are presented as mean ± SD of three independent experiments, ^*∗*^ *p* < 0.05. (f) miR-296 mimics regulated Nanog expression. J1 mESCs were transfected with mimics NC or miR-296 mimics. At 5 h after transfection, fresh medium was added and 1 *μ*M PD or an equal volume of DMSO was added to the transfected cells for another 24 h. The expression level of Nanog was detected by RT-qPCR. Error bars indicate mean ± SD of three independent experiments, ^*∗*^ *p* < 0.05 compared with controls. (g) miR-296 regulates Nanog expression. ESCs were transfected with mimics NC or miR-296 mimics for 24 h; then the protein expression level of Nanog was analyzed by western blot. Gapdh was used as a normalization control.

We found that PD treatment inhibited the expression of most miRNAs in ESCs, especially those related to ESCC family of miRNAs. More recently, we reported that ~98% of miRNAs (367 of 373) were downregulated in the CHIR-treated ESCs (GSE54145) (Table S3). This phenomenon attracted our attention and we thought that miRNAs could be almost totally inhibited in serum-free medium containing two small molecules, CHIR and PD (N2B27/2i). We then analyzed the global difference of miRNAs in these two small molecule-treated ESCs. After comparing the expression of miRNAs in CHIR- and PD-treated ESCs, we found that ~92.5% of differential miRNAs (368 of 398) were downregulated in PD and CHIR-treated ESCs. Venn diagram showed the upregulated miRNAs (Figure 5(a)) and the downregulated miRNAs (Figure 5(b)) in PD- and CHIR-treated ESCs, respectively, and the global differential miRNAs between CHIR- and PD-treated ESC are shown in Figure 5(c).

Recent reports indicate that DGCR8 can be phosphorylated by MEK/ERK, which increases its intracellular stability and induces a progrowth miRNA profile [22], while glycogen synthase kinase 3 beta phosphorylates the Drosha and increases its nuclear localization [40–42], because the phosphorylation of DGCR8 and Drosha can be repressed by PD and CHIR (Figure 5(d)), which could result in the loss of miRNAs. So most of miRNAs were inhibited in N2B27/2i ESC medium, and this result was very similar to the effect caused by the Dgcr8 knockout in ESCs. Moreover, Dgcr8 knockout ESCs were defective in differentiation even under stringent differentiation conditions (Figure 5(d)) [26]. This might be the reason that ESCs in N2B27/2i ESC medium are highly homogeneous yet fully pluripotent even in the absence of feeder, while ESCs without feeder and in the presence of LIF are flattened and heterogeneous (Figure 1(a)) [12]. Moreover, Nanog reporters are heterogeneously expressed in ESCs cultured in serum and LIF without feeder [12], and the underlying mechanism is the monoallelic expression of Nanog demonstrated by RNA fish [15]. We showed that 1 *μ*M PD treatment can change the expression of Nanog from low to high states (Figures 1(b) and 1(c)). In the meantime, miRNAs that targeted Nanog were also inhibited in PD-treated cells. For instance, RT-qPCR showed that miR-296 was significantly downregulated (Figure 4(f)).

To examine miR-296 function in PD-induced Nanog expression, we subcloned coding sequence (CDS) fragment of Nanog downstream the reporter gene in the psiCHECK-2 vector (Figure 5(e), upper panel). Luciferase assays were performed by cotransfection of the reporter vector and miR-296 mimics into 293T cells for 24 h. As shown in Figure 5(e), the reporter that harbored the CDS fragment of Nanog was significantly repressed, whereas miR-296 inhibitor could rescue luciferase activity. Furthermore, western blot and RT-qPCR showed that transfection of miR-296 mimics suppressed Nanog levels in J1 mESCs (Figures 5(f) and 5(g)); however PD can compromise miR-296 reduction on Nanog (Figure 5(f)). These results strongly suggest that PD treatment could promote Nanog expression by inhibiting the level of miRNA that targets Nanog.

## 4. Discussion

ESCs were heterogeneous because of self-activating differentiation signal of MEK/ERK that triggers differentiation of ESCs in serum-containing medium. To examine the effects of the suppression of MEK/ERK signaling to mESCs, we treated ESCs with PD and found that colonies were homogeneous in ESC morphology. GO annotation of differentially expressed genes also revealed that PD-upregulated genes were enriched for terms linked to the regulation of morphogenesis (Figure 2(c)). These results indicate that suppression of self-activating differentiation signal is positive for the homogeneous ESC morphology in serum-containing medium. Moreover, PD promoted the expression of Nanog and Klf4 under this condition (Figures 1(b) and 1(c)) and could rescue the expression of Nanog and Klf4 induced by RA (Figure 1(c)). These results indicate that PD is positive for the maintenance of the undifferentiated state of ESCs by inducing the expression of pluripotency genes and antagonizing RA-induced differentiation of ESCs.

Genome-wide expression microarray analysis confirmed these results that pluripotency-related genes were unregulated and lineage-specific markers were downregulated after PD treatment in mESCs. However, the increase of Klf4 protein level was not accompanied by that of the* Klf4* mRNA level (Figures 1(b) and 1(c)). This phenomenon indicates that MEK/ERK may regulate Klf4 expression at posttranscriptional level, consistent with previous report that MEK/ERK could phosphorylate Klf4, which results in Klf4 ubiquitination and degradation [43]. We also noted an unwarranted side effect of suppressing MEK/ERK signaling, that is, the depression of Myc messenger RNA and Myc protein levels (Figures 1(b) and 1(c)), consistent with previous study that elevated Myc is not necessary for ESC propagation [11].

The dynamical regulation of DNA methylation is important for the establishment of pluripotency in mESCs [4]. Although ESCs exist at high level of 5-hydroxymethyl cytosine (5hmC) [35], the 5hmC modification level in J1 ESCs was unchanged, even if a slight reduction (~25%) of Tet1 was caused after PD treatment (Figure 1(d)). This phenomenon might be attributed to the change of 5hmC that cannot be distinguished at the whole genome level. In addition, PD promote the expression of Prdm14, which can block mES cells from naive inner cell mass- (ICM-) like state to a primed epiblast-like state by inhibiting de novo DNA methyltransferase [38]. In undifferentiated ESCs, the majority of chromatin appears homogeneous [44]. However histone mark H3k27me3 commonly associated with repressive chromatin was not influenced by PD (Figure 1(d)).

Recently, increasing evidence suggests that miRNAs, as an important mechanism of epigenetic regulation, are crucial for normal ESC self-renewal and cellular differentiation by tightly controlling ES cell self-renewal and differentiation pathways [7]. We performed small RNA sequencing to study how miRNAs establish ESC properties in MEK/ERK pathway (Figure 3(a)). After performing fold change analysis, we noted that ~70% of miRNAs were downregulated in PD-treated samples, including the ESCC family of miRNAs. We also found that ~92.5% of differential miRNAs were downregulated after comparing the expression of miRNAs in CHIR- and PD-treated ESCs. The reduction of most miRNAs stimulated by PD and CHIR might be the reason that ESCs appear to be homogeneous in N2B27 medium supplemented with PD and CHIR. Inhibition of MEK/ERK represses Dgcr8 intracellular stability, which in turn influences miRNA profile [22]. Meanwhile, inhibition of glycogen synthase kinase 3 beta by CHIR reduces Drosha nuclear localization, which will result in the loss of miRNAs (Figure 5(d)). These could be the reasons the expression of most of miRNAs was inhibited in PD- or CHIR-treated ESCs (Figures 5(b) and 5(c)). A consistent result was also proved by GO annotation, and GO annotation revealed that PD-regulated genes were significantly enriched for terms linked to the regulation of RNA metabolic process and negative regulation of nucleic metabolic process (Figure 2(d)).

Previous study has demonstrated that Dgcr8 knockout mESCs showed a global loss of miRNAs (Figure 5(d)) and further caused proliferation defect [45]. Reintroduction of deficient canonical miRNAs that suppress inhibitors of G1-S transition can rescue the ESC proliferation defect in Dgcr8 knockout mESCs. The key factor in this process is Cdkn1a (also known as p21), an inhibitor of G1-S transition, which is inhibited by ESCC miRNAs. However, the repression of miRNAs in PD-treated ESCs did not induce the elevated p21 (Table S1), which results in proliferation defect [45], consistent with the signal transduction reporter assay that PD treatment inhibits the signaling pathway of p53 in J1 mESCs (Figure 2(f)). Thus ESC proliferation cannot be influenced by the loss of miRNAs. In addition, the monoallelic expression of Nanog that causes ESC heterogeneously in serum and LIF medium without feeder can be promoted by inhibiting the level of miRNA that targets Nanog after PD treatment (Figure 5(f)). Thus, the suppression of MEK/ERK is quite important for the homogeneous undifferentiated ESCs.

RNase III family members play diverse roles in RNA metabolism [46]. Drosha is known to play a critical role in miRNA maturation [47] and mRNA stability control [48]. For instance, in Hela cells, ~2% genes detected by Affymetrix chip were upregulated over 2-fold in Drosha-depleted cells. Furthermore, those genes are also upregulated in DGCR8-depleted cells. Thus, ~100 genes are controlled by DGCR8-Drosha complex. We cannot rule out the possibility that some of these genes indirectly influenced by CHIR and PD in N2B27/2i ESC medium exist, which in turn influence ESC pluripotency.

Taken together, our experiments showed the MEK/ERK signal-related regulation profiles and miRNAs in J1 mESCs. PD not only regulates the transcript expressions related to self-renewal and differentiation but also antagonizes the action of RA-induced differentiation. Moreover, PD was able to significantly modulate the expression of multiple miRNAs, especially those that have crucial functions in ES cell development. Thus, key regulatory genes and complex epigenetic modifications are integrated into the MEK/ERK molecular pathway, which in turn influence ES cell self-renewal and cellular differentiation.

## 5. Conclusions

ESCs have the unique ability to grow indefinitely in culture while retaining their pluripotency. This self-renewal capacity is established through the integration of several molecular pathways controlled by key regulatory genes and complex epigenetic modifications. It is reported that multiple epigenetic regulators such as miRNA and pluripotency factors can be tightly integrated into the molecular pathway and cooperate together to maintain self-renewal of ESCs. However the effects of miRNA and key regulatory genes that establish ESC properties in MEK/ERK pathway are poorly understood. In this study, we found PD-related transcripts and miRNAs that were involved in self-renewal and differentiation. We also demonstrated that PD enhances ESC self-renewal capacity not only by key regulatory genes, but also influences ES cell-specific miRNA, which in turn influences ESC self-renewal and cellular differentiation. This study also highlights that ERK1/2 signal-related miRNAs can promote ESC homogeneous.

## Supplementary Material

Detection of pluripotency of J1 mESCs by using alkaline phosphatase staining and western blot is shown in Figure 1.
Figure 2: describes pluripotency markers and signaling transduction pathways regulated by SC1.
Figure 3: describes that PD03 rescues the expression of Nanog at protein level.
Table S1: describes the differentially expressed transcripts in PD0325901 treated J1 mESCs.
Table S2: describes differentially expressed miRNAs in PD0325901 treated J1 mESCs.
Table S3: describes differentially expressed miRNAs in CHIR99021 treated J1 mESCs.
Table S4: describes primer sequences used for qPCR analyses of gene mRNAs and mature miRNAs.
