# Supplementary material for: Maintenance of Self-Renewal and Pluripotency in J1 Mouse Embryonic Stem Cells through Regulating Transcription Factor and MicroRNA Expression Induced by PD0325901
Source: Stem Cells Int. 2015 Dec 7;2016:1792573. doi: 10.1155/2016/1792573 (PMC4685126; doi:10.1155/2016/1792573)
Supplement: Supplementary file 1 — Detection of pluripotency of J1 mESCs by using alkaline phosphatase staining and western blot is shown in Figure 1. Figure 2: describes pluripotency markers and signaling transduction pathways regulated by SC1. Figure 3: describes that PD03 rescues the expression of Nanog at protein level. Table S1: describes the differentially expressed transcripts in PD0325901 treated J1 mESCs. Table S2: describes differentially expressed miRNAs in PD0325901 treated J1 mESCs. Table S3: describes differentially expressed miRNAs in CHIR99021 treated J1 mESCs. Table S4: describes primer sequences used for qPCR analyses of gene mRNAs and mature miRNAs. [file 1792573.f1.zip › 1792573.f1/mat.1792573.v2/Figure_S3_SCI_1430004.pptx]

## Slide 1
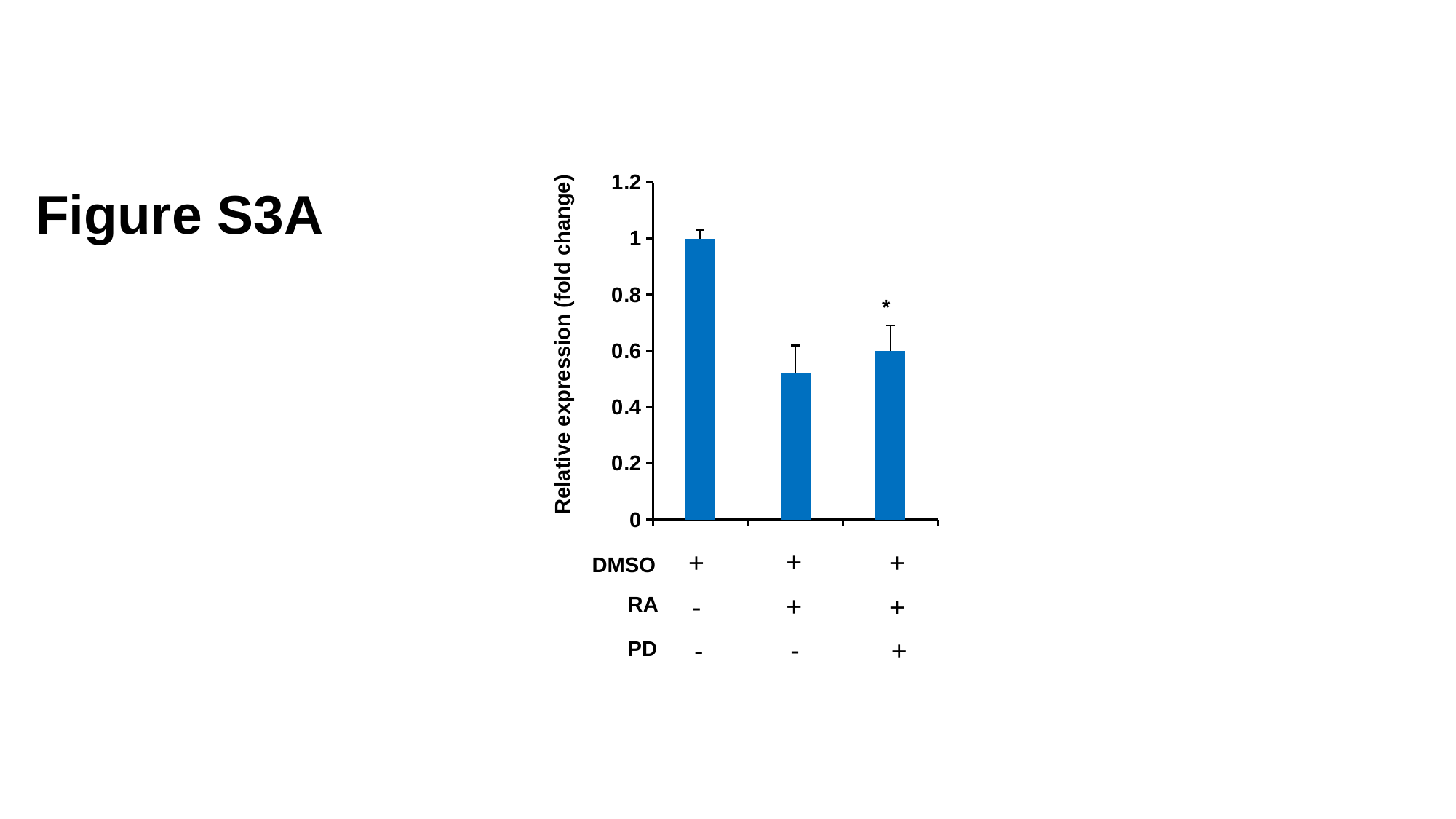

### Chart
| Category | |
|---|---|
| | 1.0 |Relative expression (fold change)
+
+
+
DMSO
+
-
+
RA
-
-
+
PD
Figure S3A

## Slide 2
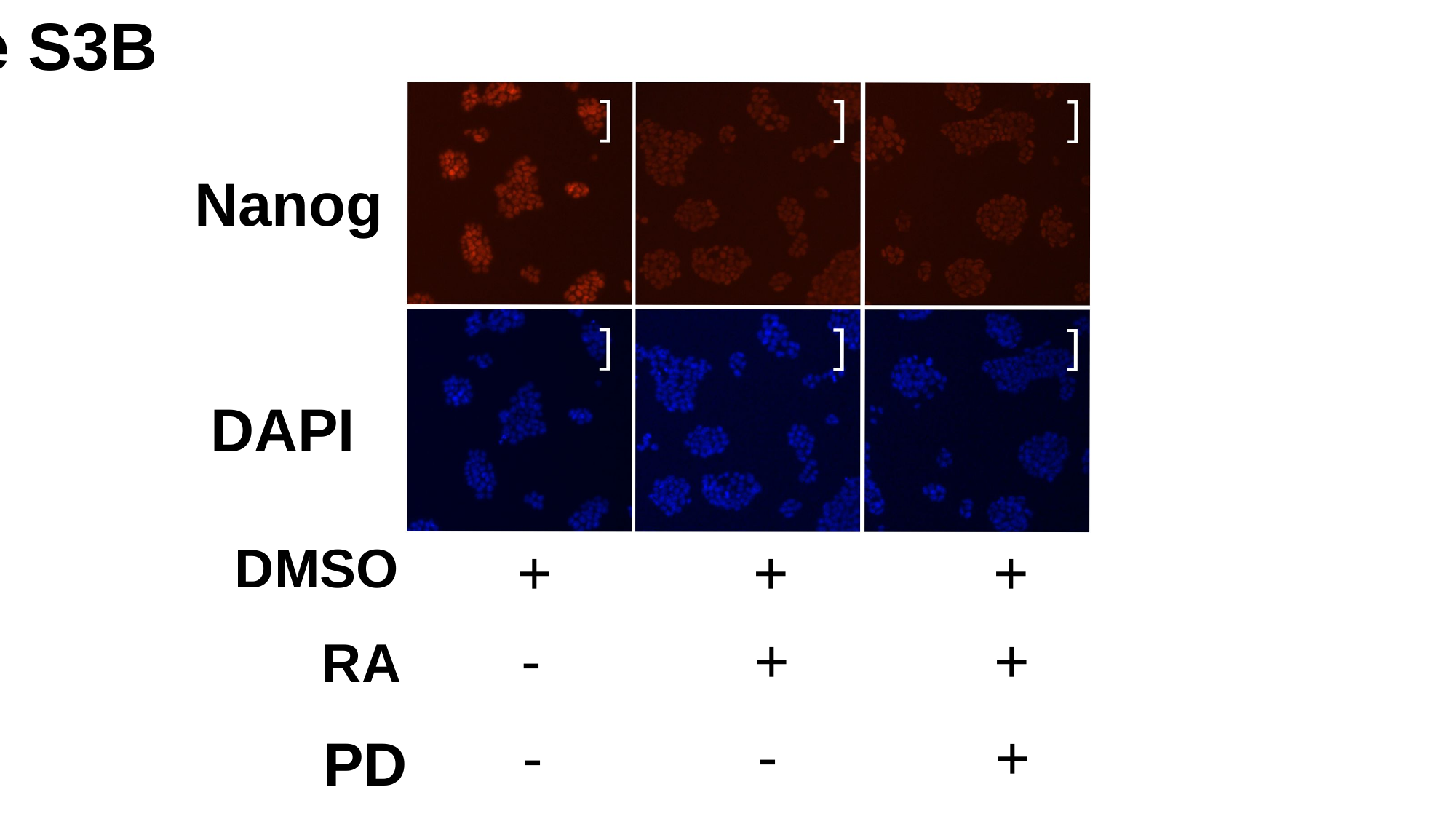

Figure S3B
Nanog
DAPI
DMSO
+
+
+
RA
-
+
+
-
-
+
PD
