# Supplementary material for: Maintenance of Self-Renewal and Pluripotency in J1 Mouse Embryonic Stem Cells through Regulating Transcription Factor and MicroRNA Expression Induced by PD0325901
Source: Stem Cells Int. 2015 Dec 7;2016:1792573. doi: 10.1155/2016/1792573 (PMC4685126; doi:10.1155/2016/1792573)
Supplement: Supplementary file 1 — Detection of pluripotency of J1 mESCs by using alkaline phosphatase staining and western blot is shown in Figure 1. Figure 2: describes pluripotency markers and signaling transduction pathways regulated by SC1. Figure 3: describes that PD03 rescues the expression of Nanog at protein level. Table S1: describes the differentially expressed transcripts in PD0325901 treated J1 mESCs. Table S2: describes differentially expressed miRNAs in PD0325901 treated J1 mESCs. Table S3: describes differentially expressed miRNAs in CHIR99021 treated J1 mESCs. Table S4: describes primer sequences used for qPCR analyses of gene mRNAs and mature miRNAs. [file 1792573.f1.zip › 1792573.f1/mat.1792573.v2/supplementary tables 432019.docx]

**Maintenance of self-renewal and pluripotency in J1 mouse embryonic stem cells through regulating transcription factor and microRNA expression induced by PD0325901**

Zhiying Ai^1,2^, Jingjing Shao^1,2^, Xinglong Shi^1,2^, Mengying Yu^2,3^, Yongyan Wu^2,3^, Juan Du^1,2^, Yong Zhang^2,3^，Zekun Guo^2,3^, ^#^

^1^College of Life Sciences, Northwest A&F University, Yangling 712100, Shaanxi, China

^2^Key Laboratory of Animal Biotechnology, Ministry of Agriculture, Northwest A&F University, Yangling 712100, Shaanxi, China

^3^College of Veterinary Medicine, Northwest A&F University, Yangling 712100, Shaanxi, China

#Correspondence: Zekun Guo

Key Laboratory of Animal Biotechnology, Ministry of Agriculture, College of Veterinary Medicine, Northwest A&F University, 3 Taicheng Road, Yangling 712100, China.

E-mail address: gzk@nwsuaf.edu.cn (Z Guo).

**Figure S1**

**
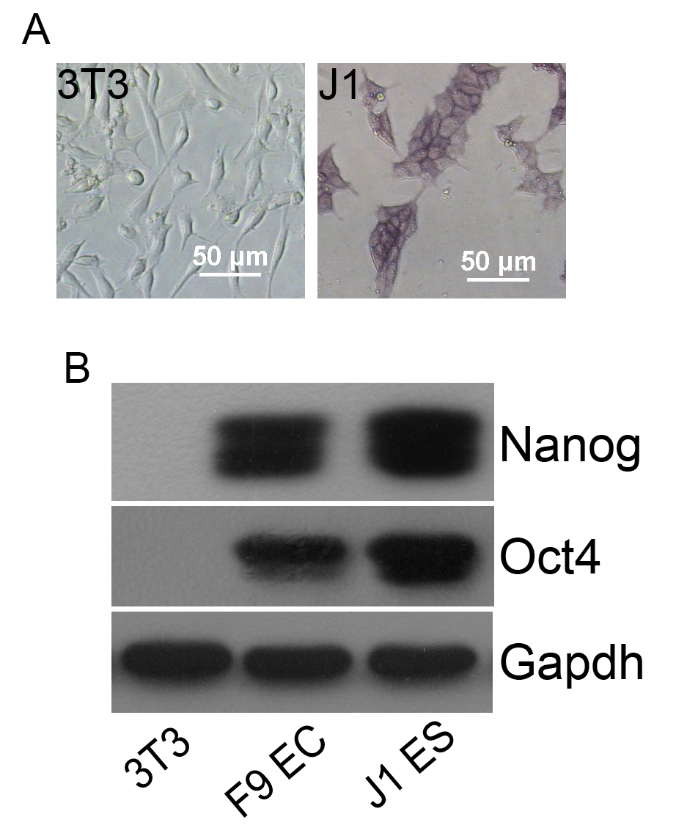
**

**Fig. S1.** J1 mESCs are pluripotent. **A**, Alkaline phosphatase staining of J1 mESC. J1 mESCs cultured in KSR/LIF without feeder-layer for two passages, After AP staining of J1 mESC colonies, morphological changes were observed and recorded under a phase contrast microscope. Scale bar = 50 μm. 3T3 cells were used for negative control. **B**, J1 mESCs express high levels of pluripotent factors. J1 mESCs cultured in KSR/LIF without feeder-layer for two passages. Then the protein expression levels of Nanog and Oct4 were analyzed by western blot. 3T3 cells and F9 ECs were used for control. Gapdh was used as a normalization control.

**Figure S2**

**
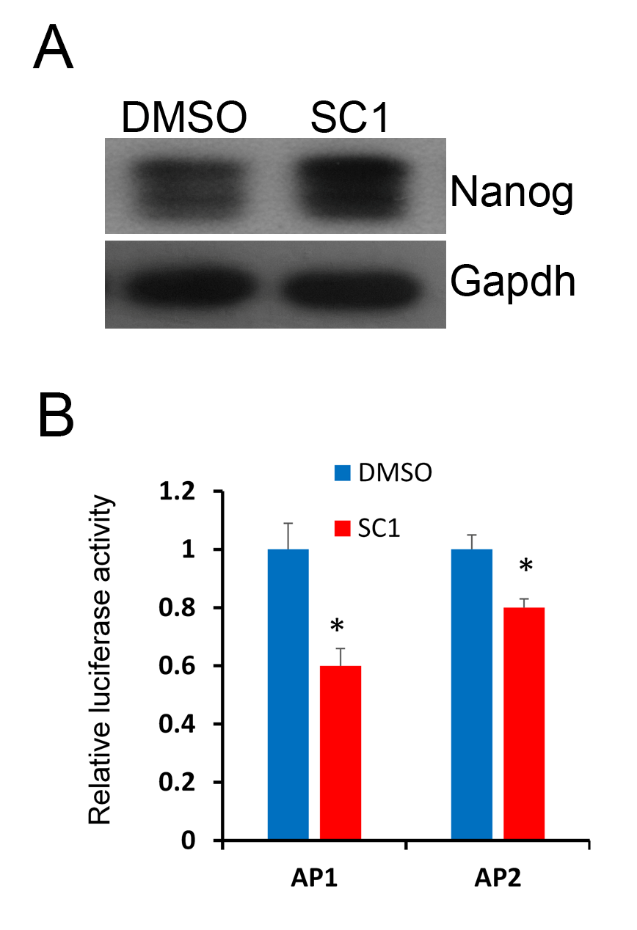
**

**Fig. S2.** Suppression of MEK/ERK signaling promotes self-renewal of J1 mESCs. **A**, SC1 regulates the expression of Nanog. J1 mESCs were treated with the 0.5 μM SC1 PD03 or equal volume DMSO for 24 h. Then the protein expression levels of Nanog was analyzed by western blot. Gapdh was used as a normalization control. B, Dual luciferase reporter assay to identify signaling transduction pathways regulated by SC1. Pathway reporter vectors (including negative control) and internal control pRL-SV40 were cotransfected by Lipofectamine 2000. 24 h after transfection, 0.5 μM SC1 or an equal volume of DMSO was added to cell medium for another 24 h. Luciferase activity is presented relative to negative control pTA-luc. Data are presented as the mean ± SD of three independent experiments, ∗, p<0.05.

**Figure S3**


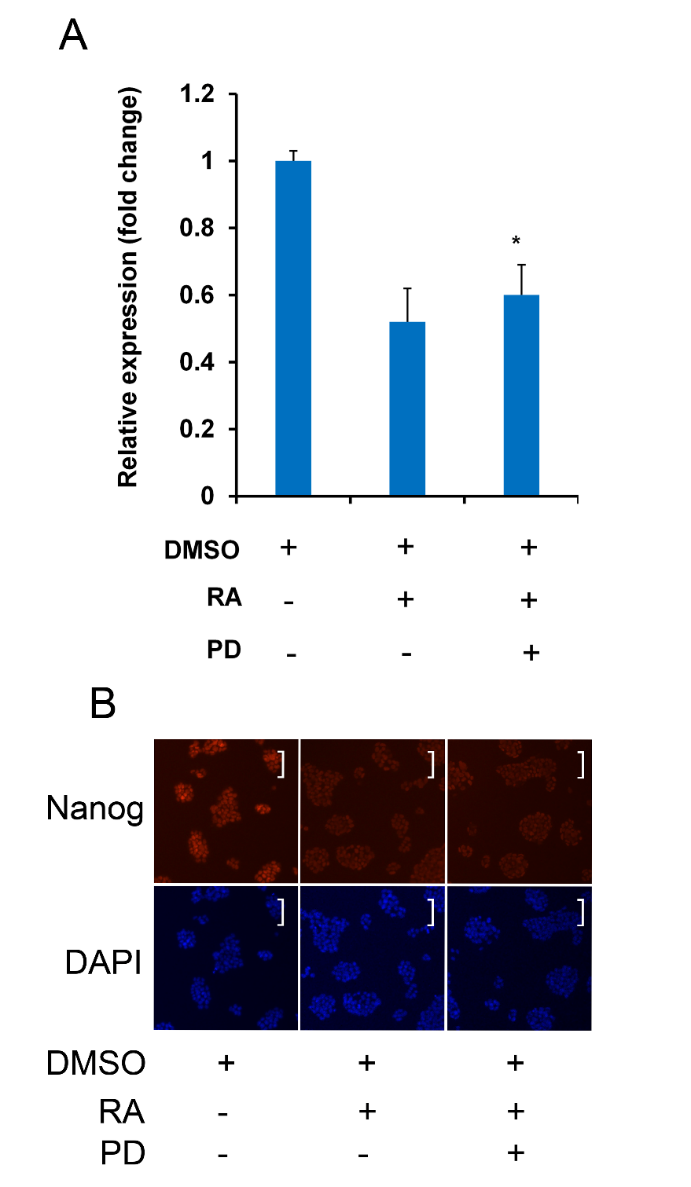


**Fig. S2.** PD03 rescues the expression of Nanog at protein level. **A**, RA induces differentiation of ESCs. ESCs were treated with 1 μM PD03 and/or together with 1 μM RA for 24 h, equal volume DMSO was added for control samples. Then the expression levels of Nanog was analyzed by Real-time RT-PCR. Gapdh was used as a normalization control. Error bars indicate mean ± SD of three independent experiments (n = 3), *, p < 0.05 compared with controls. **B**, PD03 rescues pluripotency of J1 mESCs. ESCs were treated with 1 μM PD03 and/or together with 1 μM RA for 24 h, equal volume DMSO was added for control samples. Immunofluorescence staining assay was used for analysis the expression level of Nanog. Nuclei were stained with DAPI, scale bar = 50 μm.

**Supporting information**

Additional supporting information may be found in the online version of this article at the publisher’s web site:

**Table S1.** Differentially expressed transcripts in PD0325901 treated J1 ES cells.

**Table S2.** Differentially expressed miRNAs in PD0325901 treated J1 ES cells.

**Table S3.** Differentially expressed miRNAs in CHIR99021 treated J1 ES cells.

**Table S4.** Primer sequences used for qPCR analyses of gene mRNAs and mature miRNAs.

**Supplementary Table S1. Differentially expressed transcripts in PD0325901 (PD) treated J1 ES cells. Fold change (FC) values are provided in comparison with the control ESCs which were maintained in standard ES cells medium without the addition of PD0325901.**

| GeneSymbol |  | p(PD VS J1) | |  | FC PD VS J1 |
| --- | --- | --- | --- | --- | --- |
| Bex6 |  | 0.008016 |  | | 6.282394 |
| Pramel6 |  | 0.001892 |  | | 4.798972 |
| Gm11556 |  | 3.43E-05 |  | | 4.58381 |
| Serpinb6e |  | 0.000385 |  | | 4.53956 |
| Actc1 |  | 0.004335 |  | | 4.461469 |
| Gm7747 |  | 0.000153 |  | | 4.451906 |
| Gm2903 |  | 4.47E-06 |  | | 4.168598 |
| Fbxo25 |  | 0.0079 |  | | 4.150002 |
| Gm12900 |  | 0.000182 |  | | 4.1205 |
| Arhgap19 |  | 0.000717 |  | | 4.106416 |
| LOC676811 |  | 4.18E-06 |  | | 3.932688 |
| Cox17 |  | 1.81E-05 |  | | 3.876351 |
| LOC635419 |  | 0.005681 |  | | 3.800814 |
| Snrpc |  | 0.002556 |  | | 3.749183 |
| Gm5731 |  | 1.98E-05 |  | | 3.746422 |
| C79777 |  | 0.00215 |  | | 3.741884 |
| Lyar |  | 1.87E-05 |  | | 3.711435 |
| Ly6a |  | 0.035958 |  | | 3.642174 |
| Gm2311 |  | 0.041948 |  | | 3.601306 |
| Rpl26-ps2 |  | 0.000173 |  | | 3.571956 |
| Gm6419 |  | 0.002777 |  | | 3.545507 |
| Aspa |  | 0.00154 |  | | 3.540334 |
| Cbx7 |  | 0.000106 |  | | 3.531337 |
| 1700071K01Rik |  | 0.001245 |  | | 3.53039 |
| Gm7684 |  | 0.000209 |  | | 3.508657 |
| LOC633832 |  | 6.52E-05 |  | | 3.455992 |
| Gata3 |  | 0.009461 |  | | 3.3986 |
| Serpinb6d |  | 4.39E-06 |  | | 3.366497 |
| Gm5731 |  | 1.03E-07 |  | | 3.361672 |
| Gsc |  | 0.015583 |  | | 3.310719 |
| Gm5116 |  | 7.82E-05 |  | | 3.309145 |
| Gm5080 |  | 4.05E-05 |  | | 3.306779 |
| Gm5458 |  | 1.58E-05 |  | | 3.268329 |
| Cdk1 |  | 0.000674 |  | | 3.245432 |
| Gm7966 |  | 0.003003 |  | | 3.24294 |
| Gm7686 |  | 0.000135 |  | | 3.203268 |
| Aurkb |  | 8.45E-05 |  | | 3.190786 |
| Mslnl |  | 0.004386 |  | | 3.149367 |
| Gm9372 |  | 1.12E-05 |  | | 3.137082 |
| Gm4968 |  | 0.000437 |  | | 3.107347 |
| Gm6028 |  | 0.000703 |  | | 3.040362 |
| Tlx2 |  | 4.97E-05 |  | | 2.982136 |
| Gm6632 |  | 0.012869 |  | | 2.952789 |
| Thbs1 |  | 0.020039 |  | | 2.89828 |
| Msln |  | 6.91E-06 |  | | 2.893813 |
| Tomm70a |  | 0.002876 |  | | 2.847695 |
| Gm6229 |  | 0.001732 |  | | 2.837572 |
| Hhip |  | 0.002846 |  | | 2.799614 |
| Gm5093 |  | 0.000152 |  | | 2.792027 |
| Calcoco2 |  | 4.72E-05 |  | | 2.769785 |
| Gm7575 |  | 0.000358 |  | | 2.76533 |
| Gm7219 |  | 2.89E-05 |  | | 2.747625 |
| Calcoco2 |  | 3.57E-05 |  | | 2.747446 |
| Tubb3-ps1 |  | 0.000109 |  | | 2.730294 |
| Gm5050 |  | 0.000166 |  | | 2.729259 |
| Mup-ps12 |  | 0.001172 |  | | 2.639475 |
| Gm4839 |  | 0.012049 |  | | 2.63181 |
| Xaf1 |  | 0.001342 |  | | 2.620689 |
| Gm8031 |  | 0.015993 |  | | 2.619669 |
| Gm6366 |  | 0.003195 |  | | 2.615359 |
| Gm8779 |  | 5.03E-05 |  | | 2.604248 |
| Gm13880 |  | 0.001318 |  | | 2.595612 |
| Kiss1 |  | 0.001278 |  | | 2.586443 |
| Gm7098 |  | 0.000439 |  | | 2.586111 |
| Cd3d |  | 0.009705 |  | | 2.569216 |
| Mapkap1 |  | 0.007532 |  | | 2.566379 |
| Tbc1d14 |  | 9.85E-05 |  | | 2.544516 |
| Gm12229 |  | 0.001689 |  | | 2.543504 |
| 1700016D06Rik |  | 0.002596 |  | | 2.530655 |
| Gm7167 |  | 3.09E-07 |  | | 2.530637 |
| Gm2538 |  | 0.027868 |  | | 2.529503 |
| Gm7863 |  | 0.003749 |  | | 2.529253 |
| Pramel7 |  | 0.005374 |  | | 2.524686 |
| Pa2g4 |  | 3.37E-05 |  | | 2.503804 |
| Gm7928 |  | 0.006261 |  | | 2.486704 |
| H2-M11 |  | 0.024452 |  | | 2.486086 |
| Gm7409 |  | 0.001021 |  | | 2.483668 |
| Pramef17 |  | 1.5E-05 |  | | 2.470877 |
| Ly6c1 |  | 0.001475 |  | | 2.461601 |
| Atp8b2 |  | 0.001303 |  | | 2.452176 |
| Gsdmcl1 |  | 0.001861 |  | | 2.447459 |
| Sh3rf2 |  | 0.031347 |  | | 2.44454 |
| Gm4728 |  | 0.003132 |  | | 2.443131 |
| Prl3b1 |  | 0.000489 |  | | 2.431411 |
| Gm11349 |  | 0.008417 |  | | 2.427543 |
| 4933415F23Rik |  | 0.000771 |  | | 2.393555 |
| B930086A06Rik |  | 0.000474 |  | | 2.382033 |
| Gfod1 |  | 0.001927 |  | | 2.370637 |
| Btnl1 |  | 0.03864 |  | | 2.36664 |
| LOC100046810 |  | 1.73E-05 |  | | 2.365181 |
| Lrtm2 |  | 0.038232 |  | | 2.358203 |
| 9430081H08Rik |  | 0.00762 |  | | 2.336818 |
| Xaf1 |  | 0.003313 |  | | 2.335067 |
| Sft2d1 |  | 0.005033 |  | | 2.3208 |
| LOC635091 |  | 0.001132 |  | | 2.315678 |
| Dpysl4 |  | 2.33E-05 |  | | 2.304008 |
| Zbtb7c |  | 0.008252 |  | | 2.299327 |
| Gm9880 |  | 0.000588 |  | | 2.299269 |
| Gm5317 |  | 4.4E-05 |  | | 2.281215 |
| Olfr212 |  | 0.000944 |  | | 2.275169 |
| Gm2708 |  | 0.003544 |  | | 2.273112 |
| Gm9487 |  | 0.002523 |  | | 2.259605 |
| Gm8785 |  | 0.000405 |  | | 2.251638 |
| Acta2 |  | 0.000711 |  | | 2.236824 |
| Tmprss5 |  | 0.000715 |  | | 2.229876 |
| Csf2rb2 |  | 0.002941 |  | | 2.227057 |
| Pga5 |  | 8.32E-05 |  | | 2.208263 |
| Cd248 |  | 0.003273 |  | | 2.199819 |
| Bahcc1 |  | 0.006237 |  | | 2.199398 |
| Efemp1 |  | 0.002684 |  | | 2.198857 |
| Usp15 |  | 0.000508 |  | | 2.197644 |
| Gm15303 |  | 0.002088 |  | | 2.193414 |
| Gm8662 |  | 0.000338 |  | | 2.19045 |
| LOC100040347 |  | 0.000123 |  | | 2.186815 |
| Stmn4 |  | 0.037659 |  | | 2.183435 |
| LOC100046362 |  | 0.004346 |  | | 2.175068 |
| Gm4821 |  | 0.000455 |  | | 2.163979 |
| Acta1 |  | 0.000592 |  | | 2.152112 |
| Ptpn22 |  | 0.024956 |  | | 2.151695 |
| Trim47 |  | 0.000379 |  | | 2.148025 |
| LOC100046658 |  | 0.000224 |  | | 2.145169 |
| 1200011M11Rik |  | 0.005951 |  | | 2.14295 |
| LOC100041609 |  | 0.003992 |  | | 2.139798 |
| Gm7105 |  | 0.027636 |  | | 2.137016 |
| Saa3 |  | 8.89E-05 |  | | 2.135846 |
| LOC100046061 |  | 0.00748 |  | | 2.134256 |
| Slc6a12 |  | 0.008691 |  | | 2.131998 |
| 1810013A23Rik |  | 0.017227 |  | | 2.126559 |
| Htra1 |  | 2.09E-05 |  | | 2.124265 |
| Gm826 |  | 0.019141 |  | | 2.121461 |
| 4930515G16Rik |  | 0.006009 |  | | 2.119825 |
| Osta |  | 0.000271 |  | | 2.119806 |
| LOC100046475 |  | 0.000413 |  | | 2.119284 |
| Rnft1 |  | 0.005149 |  | | 2.118556 |
| Ubxn4 |  | 0.001836 |  | | 2.117696 |
| Gm9193 |  | 0.008932 |  | | 2.116251 |
| Gm2046 |  | 0.021739 |  | | 2.113104 |
| Gm9022 |  | 0.00086 |  | | 2.113098 |
| Tsc22d2 |  | 0.022822 |  | | 2.113006 |
| Spic |  | 0.000176 |  | | 2.110397 |
| 1700003M02Rik |  | 0.003167 |  | | 2.108702 |
| Tnnc2 |  | 9.8E-05 |  | | 2.10314 |
| Gm6750 |  | 0.012195 |  | | 2.102056 |
| Sall3 |  | 0.000633 |  | | 2.101934 |
| Gm8426 |  | 0.002154 |  | | 2.100241 |
| Gm5858 |  | 0.001259 |  | | 2.089365 |
| LOC635992 |  | 0.007902 |  | | 2.070533 |
| Fhl2 |  | 0.006732 |  | | 2.067803 |
| Serpinb6c |  | 6.97E-05 |  | | 2.053327 |
| Osta |  | 4.92E-05 |  | | 2.050169 |
| Gm4804 |  | 0.001142 |  | | 2.049388 |
| Gm11701 |  | 0.001035 |  | | 2.047849 |
| Gm9697 |  | 0.000159 |  | | 2.043684 |
| Gsta2 |  | 0.037707 |  | | 2.041472 |
| Gm11701 |  | 0.002632 |  | | 2.040879 |
| Dpysl4 |  | 0.000108 |  | | 2.039511 |
| Crtac1 |  | 0.009555 |  | | 2.036826 |
| Gjb4 |  | 0.007979 |  | | 2.032797 |
| Fxyd4 |  | 0.001641 |  | | 2.032394 |
| Hesx1 |  | 0.001454 |  | | 2.028781 |
| Gm7302 |  | 0.001841 |  | | 2.026553 |
| Rasgef1a |  | 0.001734 |  | | 2.021179 |
| LOC674345 |  | 0.002325 |  | | 2.020871 |
| Gbp5 |  | 0.030234 |  | | 2.016887 |
| Gm5211 |  | 7.24E-05 |  | | 2.013968 |
| LOC100038979 |  | 0.008713 |  | | 2.013795 |
| Prss30 |  | 0.005252 |  | | 2.010471 |
| Gm3985 |  | 0.001879 |  | | 2.010249 |
| Ddr2 |  | 0.007128 |  | | 2.007661 |
| LOC100046475 |  | 0.000317 |  | | 2.004392 |
| Mras |  | 0.002155 |  | | 2.003959 |
| Gm13580 |  | 6.27E-05 |  | | 2.002639 |
| Ankmy1 |  | 0.030349 |  | | 1.994211 |
| Fst |  | 0.001559 |  | | 1.994041 |
| Cdhr1 |  | 0.002202 |  | | 1.986211 |
| LOC674914 |  | 0.000772 |  | | 1.983292 |
| Gm10144 |  | 0.001247 |  | | 1.98202 |
| Ckb |  | 1.37E-05 |  | | 1.980198 |
| Plagl1 |  | 7.07E-05 |  | | 1.977428 |
| Sdc3 |  | 0.009503 |  | | 1.977168 |
| Krt17 |  | 0.006658 |  | | 1.970984 |
| Gm11961 |  | 0.000643 |  | | 1.968508 |
| Gm9545 |  | 0.000808 |  | | 1.961617 |
| Col1a1 |  | 0.002619 |  | | 1.96052 |
| Ifitm6 |  | 0.026902 |  | | 1.958253 |
| Krt10 |  | 0.001072 |  | | 1.956307 |
| Ndfip2 |  | 0.002465 |  | | 1.953783 |
| LOC100044403 |  | 3.26E-05 |  | | 1.952488 |
| Tcl1 |  | 0.000103 |  | | 1.951318 |
| Gm12892 |  | 1.23E-05 |  | | 1.950168 |
| Msc |  | 6.81E-05 |  | | 1.949392 |
| LOC674157 |  | 1.47E-07 |  | | 1.947905 |
| Plagl1 |  | 0.000465 |  | | 1.947503 |
| Trim67 |  | 0.000425 |  | | 1.946852 |
| Sparc |  | 0.000367 |  | | 1.946134 |
| Rap1a |  | 0.004414 |  | | 1.943234 |
| C030048H21Rik |  | 0.024869 |  | | 1.942662 |
| Tnip3 |  | 0.007856 |  | | 1.942276 |
| Adamts8 |  | 0.000172 |  | | 1.939844 |
| Kcnab1 |  | 0.007384 |  | | 1.939145 |
| LOC638034 |  | 0.003072 |  | | 1.937896 |
| C81489 |  | 0.045268 |  | | 1.932548 |
| LOC100047006 |  | 0.005918 |  | | 1.931046 |
| Gm8667 |  | 0.002035 |  | | 1.930973 |
| Tmem198 |  | 0.017791 |  | | 1.930736 |
| LOC100046730 |  | 0.023749 |  | | 1.929753 |
| Uchl3 |  | 9.61E-05 |  | | 1.927952 |
| Foxd1 |  | 0.009504 |  | | 1.9262 |
| Dsp |  | 0.001355 |  | | 1.925962 |
| Gm5619 |  | 1.09E-06 |  | | 1.924629 |
| Gm4363 |  | 0.001543 |  | | 1.922868 |
| Gm4357 |  | 0.001689 |  | | 1.92283 |
| 4931407G18Rik |  | 5.56E-05 |  | | 1.92246 |
| She |  | 0.002193 |  | | 1.920737 |
| Cited2 |  | 0.000906 |  | | 1.916809 |
| Anxa1 |  | 0.000996 |  | | 1.914987 |
| Atp4b |  | 0.025017 |  | | 1.911462 |
| D13Ertd787e |  | 0.001337 |  | | 1.905735 |
| Fkbp11 |  | 7.63E-06 |  | | 1.904132 |
| Gm6949 |  | 7.51E-05 |  | | 1.896686 |
| Gm4858 |  | 4.17E-05 |  | | 1.89651 |
| Mme |  | 0.000285 |  | | 1.895239 |
| Agpat9 |  | 0.0032 |  | | 1.894898 |
| LOC100047210 |  | 0.024718 |  | | 1.891033 |
| Gm7810 |  | 0.001081 |  | | 1.889606 |
| LOC100041777 |  | 0.007281 |  | | 1.888684 |
| Dyrk1b |  | 7.27E-05 |  | | 1.888165 |
| 3830417A13Rik |  | 0.021587 |  | | 1.884399 |
| Kbtbd11 |  | 0.00013 |  | | 1.883959 |
| 1700025K04Rik |  | 0.035323 |  | | 1.883179 |
| A930001C03Rik |  | 0.004604 |  | | 1.882112 |
| LOC100048515 |  | 0.001121 |  | | 1.880696 |
| Gm12209 |  | 0.036641 |  | | 1.878465 |
| 8030402F09Rik |  | 0.00327 |  | | 1.877715 |
| Gm2486 |  | 0.001308 |  | | 1.875622 |
| Cd5 |  | 0.00985 |  | | 1.873989 |
| Anxa1 |  | 0.014942 |  | | 1.873758 |
| Gm9012 |  | 0.030935 |  | | 1.873511 |
| Mme |  | 0.00025 |  | | 1.872641 |
| Sly |  | 0.016788 |  | | 1.868895 |
| C79240 |  | 0.013463 |  | | 1.868045 |
| Gm10639 |  | 0.017052 |  | | 1.86799 |
| Hck |  | 0.000439 |  | | 1.867675 |
| BB287469 |  | 0.000356 |  | | 1.866041 |
| Gm7109 |  | 0.000628 |  | | 1.863428 |
| 2810408P10Rik |  | 0.015898 |  | | 1.858229 |
| Ckb |  | 0.000164 |  | | 1.854084 |
| D630045M09Rik |  | 1.75E-05 |  | | 1.852663 |
| 4931402H11Rik |  | 0.000619 |  | | 1.851793 |
| LOC100047590 |  | 0.027539 |  | | 1.851238 |
| Zscan4-ps1 |  | 0.000536 |  | | 1.847604 |
| LOC100046158 |  | 0.021881 |  | | 1.847014 |
| Olfr53 |  | 0.007499 |  | | 1.845944 |
| Tcfcp2l1 |  | 0.000766 |  | | 1.845704 |
| Gm4862 |  | 0.00011 |  | | 1.844487 |
| Cd44 |  | 0.000912 |  | | 1.844104 |
| Aox3 |  | 0.025605 |  | | 1.843722 |
| LOC634916 |  | 0.006305 |  | | 1.842674 |
| Mdga1 |  | 0.011986 |  | | 1.840814 |
| LOC641136 |  | 0.000682 |  | | 1.840255 |
| Pla2g16 |  | 8.5E-05 |  | | 1.840221 |
| Emp1 |  | 0.000123 |  | | 1.839472 |
| Teddm1 |  | 0.005442 |  | | 1.839292 |
| Mageb16-ps1 |  | 0.000128 |  | | 1.838397 |
| Prdm14 |  | 0.000198 |  | | 1.835282 |
| Klk11 |  | 0.00436 |  | | 1.834874 |
| Hecw2 |  | 0.000507 |  | | 1.834543 |
| Gm5860 |  | 0.008378 |  | | 1.834519 |
| Ly6c1 |  | 0.003247 |  | | 1.832279 |
| Rdh1 |  | 0.008232 |  | | 1.832267 |
| Cyp2j7-ps |  | 0.003346 |  | | 1.830558 |
| Clec4a1 |  | 0.034244 |  | | 1.82516 |
| Casd1 |  | 0.000851 |  | | 1.824993 |
| Rps27a-ps1 |  | 0.026127 |  | | 1.824832 |
| Vmn1r45 |  | 0.01073 |  | | 1.824704 |
| Gsta3 |  | 0.000265 |  | | 1.822743 |
| Gm11938 |  | 0.00755 |  | | 1.821174 |
| Cr2 |  | 0.00106 |  | | 1.820736 |
| LOC100044696 |  | 0.021094 |  | | 1.81967 |
| Lman1l |  | 0.001591 |  | | 1.819022 |
| Gucy2e |  | 0.006082 |  | | 1.818798 |
| Casq2 |  | 0.032885 |  | | 1.816069 |
| Nmur2 |  | 0.028747 |  | | 1.814144 |
| AU015836 |  | 0.00522 |  | | 1.811049 |
| Cnr2 |  | 5.04E-05 |  | | 1.806741 |
| AW060742 |  | 0.000555 |  | | 1.805138 |
| Vax2os2 |  | 0.00248 |  | | 1.803575 |
| Mitf |  | 0.018211 |  | | 1.800518 |
| Gm2761 |  | 0.000436 |  | | 1.795798 |
| Prss35 |  | 0.00256 |  | | 1.793398 |
| Fam169b |  | 0.000781 |  | | 1.791913 |
| Tnfaip6 |  | 0.033692 |  | | 1.790383 |
| 1700020C07Rik |  | 0.016264 |  | | 1.788472 |
| LOC100044649 |  | 0.003281 |  | | 1.784012 |
| Vmn2r-ps43 |  | 0.002993 |  | | 1.7836 |
| 4930568K20Rik |  | 0.004904 |  | | 1.783164 |
| Pou4f2 |  | 0.00064 |  | | 1.782708 |
| Sema3c |  | 0.003481 |  | | 1.781557 |
| Prlr |  | 0.002243 |  | | 1.781411 |
| Olfr577 |  | 0.019001 |  | | 1.780867 |
| Olfr951 |  | 0.00901 |  | | 1.780773 |
| Prdm1 |  | 0.005997 |  | | 1.779447 |
| Defa-rs2 |  | 0.005251 |  | | 1.777679 |
| LOC654469 |  | 7.98E-05 |  | | 1.775984 |
| Gm3950 |  | 0.001238 |  | | 1.775976 |
| Cyp4a31 |  | 0.02729 |  | | 1.775911 |
| Myl9 |  | 8.27E-05 |  | | 1.774125 |
| D630039A03Rik |  | 0.008655 |  | | 1.77169 |
| 1700025L06Rik |  | 0.000524 |  | | 1.771563 |
| Gm9573 |  | 0.00225 |  | | 1.765492 |
| Abca17 |  | 0.001877 |  | | 1.76451 |
| Nuak2 |  | 0.001696 |  | | 1.762406 |
| Pdgfc |  | 2.34E-05 |  | | 1.762383 |
| Sox7 |  | 0.0008 |  | | 1.760962 |
| Zfp386 |  | 0.000358 |  | | 1.758723 |
| Dsp |  | 0.000154 |  | | 1.757514 |
| 4930593A02Rik |  | 0.000157 |  | | 1.757241 |
| Frs3 |  | 0.005466 |  | | 1.75642 |
| Gm8300 |  | 0.004304 |  | | 1.755756 |
| Gfra2 |  | 0.001838 |  | | 1.754919 |
| Ngfr |  | 0.000776 |  | | 1.754813 |
| E130304I02Rik |  | 0.004522 |  | | 1.754587 |
| Ntrk2 |  | 0.000964 |  | | 1.754046 |
| P4ha1 |  | 0.009809 |  | | 1.753135 |
| Gm7644 |  | 0.019913 |  | | 1.751 |
| Gm2049 |  | 0.000145 |  | | 1.750334 |
| Gm2908 |  | 0.026794 |  | | 1.749188 |
| Gm5039 |  | 0.023925 |  | | 1.749154 |
| Pmp22 |  | 0.003294 |  | | 1.747879 |
| 4931407E12Rik |  | 0.00021 |  | | 1.746864 |
| LOC100046976 |  | 0.001888 |  | | 1.742683 |
| Sfrp1 |  | 0.00017 |  | | 1.742035 |
| LOC641136 |  | 0.006701 |  | | 1.741794 |
| Lrrc34 |  | 0.000539 |  | | 1.741303 |
| Ncam1 |  | 0.001737 |  | | 1.740512 |
| Ttn |  | 0.01142 |  | | 1.737706 |
| LOC432569 |  | 0.002529 |  | | 1.736499 |
| Gm2479 |  | 0.014493 |  | | 1.734818 |
| Gm4157 |  | 2.76E-05 |  | | 1.734497 |
| Gm14277 |  | 0.000468 |  | | 1.733927 |
| Mdfic |  | 0.000901 |  | | 1.732504 |
| Pkib |  | 0.000123 |  | | 1.731473 |
| LOC100047306 |  | 0.006892 |  | | 1.73093 |
| 4930581F22Rik |  | 0.000377 |  | | 1.729534 |
| Tagln |  | 0.002483 |  | | 1.729208 |
| Ahnak |  | 0.000364 |  | | 1.727905 |
| Hes1 |  | 0.006353 |  | | 1.727806 |
| Tubb3 |  | 0.00024 |  | | 1.726913 |
| Smyd1 |  | 4.1E-05 |  | | 1.726531 |
| Tpst1 |  | 0.002307 |  | | 1.726419 |
| Pcolce2 |  | 0.000236 |  | | 1.723292 |
| Nhlh1 |  | 0.004344 |  | | 1.722683 |
| Gm6189 |  | 0.003177 |  | | 1.721332 |
| Gm5316 |  | 0.000419 |  | | 1.719575 |
| 5730588L14Rik |  | 0.016062 |  | | 1.715521 |
| Ifitm1 |  | 0.000239 |  | | 1.715406 |
| Tspan32 |  | 0.000503 |  | | 1.71435 |
| Chst3 |  | 0.000649 |  | | 1.712073 |
| Gm13807 |  | 0.001053 |  | | 1.711312 |
| Prh1 |  | 0.015595 |  | | 1.711189 |
| Gm6003 |  | 0.00105 |  | | 1.710857 |
| Zfp345 |  | 0.005827 |  | | 1.710248 |
| 1700017I07Rik |  | 0.018078 |  | | 1.709167 |
| 5730408K05Rik |  | 0.002295 |  | | 1.709057 |
| Wisp1 |  | 0.034545 |  | | 1.70859 |
| A030001D16Rik |  | 0.02227 |  | | 1.707716 |
| LOC100044381 |  | 0.000501 |  | | 1.706983 |
| A530047J11Rik |  | 0.000307 |  | | 1.705498 |
| Tgm1 |  | 0.004592 |  | | 1.704877 |
| Gm4026 |  | 0.015831 |  | | 1.703311 |
| Gm9100 |  | 0.007277 |  | | 1.702492 |
| Gm14340 |  | 0.003541 |  | | 1.701642 |
| Gm16367 |  | 0.010435 |  | | 1.6993 |
| AA763521 |  | 0.006556 |  | | 1.697055 |
| Uncx |  | 0.002534 |  | | 1.696605 |
| Gm2486 |  | 0.000771 |  | | 1.696283 |
| Tmem174 |  | 0.008239 |  | | 1.69542 |
| Pcdha8 |  | 0.013144 |  | | 1.695167 |
| D5Wsu152e |  | 7.42E-05 |  | | 1.69388 |
| Gm11496 |  | 0.000139 |  | | 1.693732 |
| Mtf2 |  | 3.82E-05 |  | | 1.691563 |
| Gm8705 |  | 0.026825 |  | | 1.690864 |
| Epb4.9 |  | 0.002961 |  | | 1.689561 |
| 8430439B09Rik |  | 0.020337 |  | | 1.687782 |
| Gm7765 |  | 0.001605 |  | | 1.687179 |
| Krt9 |  | 0.015253 |  | | 1.686551 |
| Alppl2 |  | 0.0075 |  | | 1.686391 |
| Padi2 |  | 0.002144 |  | | 1.68559 |
| Mpl |  | 0.000884 |  | | 1.685139 |
| Dpysl3 |  | 0.001048 |  | | 1.685004 |
| Gm15550 |  | 0.003544 |  | | 1.684668 |
| Pex26 |  | 0.005387 |  | | 1.683743 |
| Padi2 |  | 3.4E-06 |  | | 1.682971 |
| Cga |  | 0.001021 |  | | 1.682133 |
| Syt1 |  | 0.023218 |  | | 1.681888 |
| EG638052 |  | 0.002603 |  | | 1.681886 |
| AU015836 |  | 0.006776 |  | | 1.680951 |
| Mdfic |  | 0.02039 |  | | 1.680659 |
| 3010033K07Rik |  | 0.022995 |  | | 1.680529 |
| Csn1s2b |  | 0.016985 |  | | 1.679161 |
| Casc1 |  | 0.008385 |  | | 1.679073 |
| Ctdsp2 |  | 0.002547 |  | | 1.678534 |
| Samd9l |  | 0.005767 |  | | 1.678395 |
| LOC100047557 |  | 0.006034 |  | | 1.678386 |
| LOC630152 |  | 0.006093 |  | | 1.677203 |
| Gm6787 |  | 0.005229 |  | | 1.676973 |
| Atp8b4 |  | 0.011658 |  | | 1.675354 |
| LOC100048531 |  | 0.002402 |  | | 1.675081 |
| Slc16a12 |  | 0.020608 |  | | 1.674935 |
| Nkain4 |  | 0.000582 |  | | 1.673567 |
| A830073O21Rik |  | 6.17E-05 |  | | 1.672676 |
| Dleu2 |  | 0.001725 |  | | 1.671013 |
| Abpe |  | 0.037069 |  | | 1.668096 |
| Gm2891 |  | 0.007403 |  | | 1.667638 |
| Nid1 |  | 0.000221 |  | | 1.667562 |
| Gm2219 |  | 0.000334 |  | | 1.667152 |
| Ifitm3 |  | 0.001323 |  | | 1.666035 |
| Adamts8 |  | 0.001096 |  | | 1.665542 |
| A230101C19Rik |  | 0.024017 |  | | 1.66554 |
| Ttn |  | 0.002574 |  | | 1.664562 |
| Tgfbi |  | 0.000675 |  | | 1.663279 |
| LOC100045064 |  | 0.000619 |  | | 1.66048 |
| Tcfcp2l1 |  | 0.003117 |  | | 1.659771 |
| Ccl2 |  | 0.004093 |  | | 1.659743 |
| Gm9041 |  | 0.031734 |  | | 1.659616 |
| Gm7676 |  | 0.001153 |  | | 1.659545 |
| Mapt |  | 0.000784 |  | | 1.659209 |
| E230006M18Rik |  | 0.001562 |  | | 1.658578 |
| Coro2b |  | 0.000556 |  | | 1.65847 |
| Lrrc15 |  | 0.000429 |  | | 1.656626 |
| Gm2797 |  | 0.008136 |  | | 1.655775 |
| Nanog |  | 0.000317 |  | | 1.655508 |
| Gm15616 |  | 7.83E-05 |  | | 1.654226 |
| Srgap3 |  | 0.000209 |  | | 1.653114 |
| Pld1 |  | 3.04E-05 |  | | 1.65193 |
| Klk10 |  | 0.000372 |  | | 1.651315 |
| Scn5a |  | 0.001852 |  | | 1.650828 |
| Gm14431 |  | 0.000874 |  | | 1.649928 |
| 4930525G20Rik |  | 0.000118 |  | | 1.64946 |
| Npm3 |  | 0.002399 |  | | 1.648884 |
| LOC672978 |  | 0.000939 |  | | 1.648825 |
| Gm3548 |  | 0.000232 |  | | 1.648704 |
| Lce1a2 |  | 5.55E-05 |  | | 1.646526 |
| Gm5604 |  | 0.000273 |  | | 1.646348 |
| Gm6209 |  | 0.003132 |  | | 1.644724 |
| Pde3a |  | 0.003547 |  | | 1.644057 |
| Elf3 |  | 0.000175 |  | | 1.643988 |
| Krtap10-10 |  | 0.000272 |  | | 1.643673 |
| Cd244 |  | 0.005851 |  | | 1.643091 |
| Gm2016 |  | 0.000488 |  | | 1.641936 |
| Ccl25 |  | 0.004428 |  | | 1.640961 |
| Atp9a |  | 0.000598 |  | | 1.639742 |
| 1700042O10Rik |  | 0.001568 |  | | 1.639619 |
| 8430406I07Rik |  | 0.000496 |  | | 1.63885 |
| 4930519A11Rik |  | 0.005966 |  | | 1.637932 |
| Rhobtb1 |  | 0.001164 |  | | 1.637572 |
| Mras |  | 0.000673 |  | | 1.637528 |
| Gm9102 |  | 0.007005 |  | | 1.63724 |
| Lgals3 |  | 0.000115 |  | | 1.637206 |
| Wbscr28 |  | 0.009815 |  | | 1.636286 |
| Gpr45 |  | 0.049251 |  | | 1.635468 |
| Zfp600 |  | 0.015076 |  | | 1.635417 |
| LOC677608 |  | 5.62E-06 |  | | 1.634799 |
| LOC633654 |  | 0.000458 |  | | 1.633436 |
| Fxyd1 |  | 0.005009 |  | | 1.633192 |
| 2310043M15Rik |  | 0.000163 |  | | 1.633116 |
| Kcnq1ot1 |  | 0.04505 |  | | 1.631918 |
| Slc6a1 |  | 0.021819 |  | | 1.631829 |
| Zfhx2 |  | 0.002302 |  | | 1.629693 |
| Ksr2 |  | 0.001136 |  | | 1.628458 |
| Nanogpd |  | 1.21E-05 |  | | 1.628219 |
| Folr2 |  | 0.000539 |  | | 1.627803 |
| Apobec2 |  | 0.017774 |  | | 1.627208 |
| Gm15698 |  | 0.004034 |  | | 1.626832 |
| Chst8 |  | 0.000251 |  | | 1.626601 |
| 8030411F24Rik |  | 0.03086 |  | | 1.626393 |
| Ttll10 |  | 0.000854 |  | | 1.626331 |
| Duox1 |  | 0.006188 |  | | 1.626013 |
| AW050198 |  | 0.006848 |  | | 1.625941 |
| 4930547M16Rik |  | 0.041781 |  | | 1.625104 |
| Krt14 |  | 0.00995 |  | | 1.624142 |
| Gm3350 |  | 0.001972 |  | | 1.622756 |
| Myo1f |  | 0.002125 |  | | 1.622426 |
| Spag6 |  | 0.002324 |  | | 1.621646 |
| Dnalc1 |  | 0.014144 |  | | 1.621382 |
| S100a5 |  | 0.024597 |  | | 1.620845 |
| LOC432754 |  | 0.046774 |  | | 1.619899 |
| LOC100044394 |  | 0.000118 |  | | 1.619739 |
| Dpysl3 |  | 0.001866 |  | | 1.619705 |
| Zfp72 |  | 0.005268 |  | | 1.616577 |
| 1700030N03Rik |  | 0.00164 |  | | 1.616205 |
| 2900072G11Rik |  | 0.004522 |  | | 1.616142 |
| 2300005B03Rik |  | 0.001387 |  | | 1.615128 |
| Mrgprf |  | 0.002469 |  | | 1.614037 |
| Siglece |  | 0.00066 |  | | 1.613334 |
| Gm5478 |  | 0.006739 |  | | 1.613123 |
| Klk1b26 |  | 0.002007 |  | | 1.612874 |
| LOC100048698 |  | 0.000552 |  | | 1.612732 |
| Eln |  | 0.027239 |  | | 1.612208 |
| Zfp788 |  | 0.008651 |  | | 1.611559 |
| 6230415J03Rik |  | 0.008485 |  | | 1.611489 |
| Gm2312 |  | 0.017524 |  | | 1.611211 |
| Cdan1 |  | 0.008465 |  | | 1.611137 |
| Ephx3 |  | 0.00123 |  | | 1.611116 |
| Olfr1350 |  | 0.018698 |  | | 1.609284 |
| LOC100043999 |  | 0.00071 |  | | 1.609096 |
| Gpc4 |  | 0.002833 |  | | 1.608904 |
| LOC677262 |  | 0.000137 |  | | 1.608346 |
| Btnl1 |  | 0.002528 |  | | 1.607861 |
| S100a16 |  | 0.00029 |  | | 1.607681 |
| Gm2571 |  | 6.49E-05 |  | | 1.605555 |
| Alox12b |  | 0.014824 |  | | 1.60459 |
| Capn6 |  | 0.03418 |  | | 1.604181 |
| Nox1 |  | 0.010949 |  | | 1.603665 |
| Mitf |  | 2.01E-05 |  | | 1.603271 |
| Olfr1341 |  | 0.001293 |  | | 1.603215 |
| Tas2r109 |  | 0.001286 |  | | 1.602968 |
| Gm5595 |  | 0.005709 |  | | 1.602812 |
| Hspa1l |  | 0.030003 |  | | 1.60221 |
| BC024139 |  | 0.016364 |  | | 1.601526 |
| Cryab |  | 0.006431 |  | | 1.601518 |
| 5830468F06Rik |  | 0.009438 |  | | 1.601034 |
| Ckmt1 |  | 5.59E-05 |  | | 1.60099 |
| Rgs7 |  | 0.003903 |  | | 1.600908 |
| Gm4633 |  | 0.00281 |  | | 1.600458 |
| Frmd4a |  | 0.001519 |  | | 1.599316 |
| AI450886 |  | 0.022128 |  | | 1.599282 |
| Gm6257 |  | 0.001556 |  | | 1.599208 |
| Btn2a2 |  | 0.030876 |  | | 1.598524 |
| Adam5 |  | 0.025924 |  | | 1.598391 |
| Sfrp5 |  | 0.001454 |  | | 1.598224 |
| Nos2 |  | 0.000547 |  | | 1.597004 |
| 4930554C24Rik |  | 0.01986 |  | | 1.596748 |
| Ubd |  | 0.00281 |  | | 1.596276 |
| Vmn1r216 |  | 0.017381 |  | | 1.595634 |
| 2610528A11Rik |  | 0.010233 |  | | 1.594855 |
| Lrrc31 |  | 0.000442 |  | | 1.594301 |
| Tm6sf2 |  | 0.001519 |  | | 1.593969 |
| Gm11543 |  | 0.029234 |  | | 1.593386 |
| Irx6 |  | 0.006453 |  | | 1.592035 |
| Zfp282 |  | 0.004136 |  | | 1.591422 |
| Gm14410 |  | 0.000912 |  | | 1.590993 |
| Aplnr |  | 0.022816 |  | | 1.590518 |
| LOC100044611 |  | 0.007972 |  | | 1.590389 |
| LOC100044755 |  | 0.003206 |  | | 1.589997 |
| Krt73 |  | 0.000189 |  | | 1.588732 |
| Gm2692 |  | 0.00822 |  | | 1.588276 |
| Gm7767 |  | 0.007779 |  | | 1.588235 |
| Lrrn2 |  | 0.001247 |  | | 1.587901 |
| Gpr133 |  | 0.013935 |  | | 1.58615 |
| Galnt14 |  | 0.035092 |  | | 1.586001 |
| Runx1t1 |  | 0.000785 |  | | 1.585616 |
| Slco2a1 |  | 0.004119 |  | | 1.585417 |
| Rnf180 |  | 0.002586 |  | | 1.585286 |
| Gvin1 |  | 0.025867 |  | | 1.584323 |
| Gm9519 |  | 0.041503 |  | | 1.583651 |
| Prrt1 |  | 0.009222 |  | | 1.583618 |
| Itsn1 |  | 0.01399 |  | | 1.583511 |
| Spp1 |  | 0.004714 |  | | 1.583389 |
| Myo1f |  | 0.000767 |  | | 1.582206 |
| Kng1 |  | 0.000122 |  | | 1.581848 |
| Gm8719 |  | 0.003182 |  | | 1.580511 |
| Pgpep1l |  | 0.024986 |  | | 1.580211 |
| Gm12409 |  | 0.017118 |  | | 1.57854 |
| Ptch1 |  | 0.000228 |  | | 1.578359 |
| Zfp790 |  | 0.003935 |  | | 1.576828 |
| Vmn2r90 |  | 0.007598 |  | | 1.576739 |
| B230334C09Rik |  | 0.006256 |  | | 1.576723 |
| Lamc2 |  | 3.32E-05 |  | | 1.575155 |
| Gm4902 |  | 0.000328 |  | | 1.575068 |
| 4931417G12Rik |  | 0.001444 |  | | 1.574946 |
| Cxcr7 |  | 0.002332 |  | | 1.574206 |
| Gm13251 |  | 0.000443 |  | | 1.574006 |
| Wwp1 |  | 0.000582 |  | | 1.57286 |
| Tmem8c |  | 0.001857 |  | | 1.572527 |
| Tspan32 |  | 0.011724 |  | | 1.572158 |
| Spink3 |  | 0.00221 |  | | 1.571138 |
| Csnk2a1-ps |  | 0.008166 |  | | 1.571124 |
| LOC621118 |  | 0.010026 |  | | 1.570652 |
| 4931431F19Rik |  | 0.005639 |  | | 1.570378 |
| Sp3 |  | 0.000139 |  | | 1.568533 |
| Gm13430 |  | 0.001606 |  | | 1.56834 |
| Wfdc3 |  | 0.008608 |  | | 1.568324 |
| Mup20 |  | 0.002069 |  | | 1.568206 |
| Acyp2 |  | 0.002152 |  | | 1.567996 |
| Cdkl1 |  | 0.031202 |  | | 1.56789 |
| Reg3d |  | 0.000952 |  | | 1.567313 |
| Cyp2b10 |  | 0.000679 |  | | 1.566263 |
| Gdf15 |  | 0.000594 |  | | 1.565895 |
| Gm6970 |  | 0.000243 |  | | 1.56583 |
| LOC631980 |  | 0.034663 |  | | 1.564624 |
| Serpinb1a |  | 0.026933 |  | | 1.564053 |
| Gm2503 |  | 6.6E-05 |  | | 1.56339 |
| Aff4 |  | 0.005722 |  | | 1.562501 |
| Pabpc5 |  | 0.002598 |  | | 1.562167 |
| LOC100048731 |  | 0.015118 |  | | 1.561849 |
| Airn |  | 2.35E-05 |  | | 1.561721 |
| Gm8960 |  | 0.002621 |  | | 1.561371 |
| Myl3 |  | 0.015743 |  | | 1.561278 |
| Lst1 |  | 4.09E-05 |  | | 1.56106 |
| Cst13 |  | 0.000981 |  | | 1.560968 |
| Stk30 |  | 0.000584 |  | | 1.56094 |
| Ahnak |  | 0.00105 |  | | 1.560001 |
| Lmx1b |  | 0.015431 |  | | 1.558916 |
| Hoxd13 |  | 0.005343 |  | | 1.558848 |
| LOC100045919 |  | 0.000417 |  | | 1.558692 |
| Jam2 |  | 0.00037 |  | | 1.558632 |
| Iqcd |  | 0.000168 |  | | 1.557198 |
| Sox21 |  | 0.000648 |  | | 1.557038 |
| Gm10318 |  | 0.001326 |  | | 1.556321 |
| Ifna12 |  | 0.008141 |  | | 1.555751 |
| Lce1a2 |  | 0.000289 |  | | 1.555319 |
| LOC632173 |  | 0.003231 |  | | 1.554449 |
| Gzma |  | 0.041188 |  | | 1.554285 |
| Olfr144 |  | 0.004034 |  | | 1.553801 |
| Tubb2b |  | 0.000635 |  | | 1.553567 |
| BC048507 |  | 0.000199 |  | | 1.553352 |
| Ppp2r2c |  | 0.002075 |  | | 1.553236 |
| C77847 |  | 0.000377 |  | | 1.552889 |
| LOC676890 |  | 0.001988 |  | | 1.552347 |
| Acyp1 |  | 0.027734 |  | | 1.552304 |
| LOC100048885 |  | 0.005006 |  | | 1.55191 |
| BC064078 |  | 0.005363 |  | | 1.55167 |
| Rhcg |  | 0.008433 |  | | 1.551542 |
| Gm6242 |  | 3.94E-05 |  | | 1.551513 |
| Gm2022 |  | 0.005307 |  | | 1.551072 |
| Gm7702 |  | 0.01101 |  | | 1.549545 |
| Pclo |  | 0.000486 |  | | 1.549137 |
| Gm2046 |  | 0.005668 |  | | 1.54801 |
| Gm3115 |  | 0.002458 |  | | 1.546714 |
| Prss53 |  | 0.005275 |  | | 1.546366 |
| LOC100044477 |  | 0.002543 |  | | 1.546107 |
| Abcb1b |  | 3.99E-06 |  | | 1.545425 |
| Krtap20-2 |  | 0.013421 |  | | 1.545262 |
| Olfr190 |  | 0.028769 |  | | 1.544833 |
| Cyp4a29 |  | 6.64E-05 |  | | 1.544305 |
| Cabp4 |  | 0.038028 |  | | 1.543993 |
| Slco4c1 |  | 0.003727 |  | | 1.543299 |
| Gm428 |  | 0.01543 |  | | 1.543299 |
| Gm5106 |  | 0.004248 |  | | 1.542962 |
| Gm6947 |  | 0.028735 |  | | 1.542885 |
| Ankrd43 |  | 0.024787 |  | | 1.542509 |
| Wdfy1 |  | 0.000983 |  | | 1.541522 |
| Gsta3 |  | 1.36E-05 |  | | 1.541348 |
| Kirrel3 |  | 0.011089 |  | | 1.541328 |
| Mapt |  | 0.000738 |  | | 1.541262 |
| Gm2976 |  | 0.032824 |  | | 1.540946 |
| Kcns3 |  | 0.000311 |  | | 1.54056 |
| Lama3 |  | 0.000103 |  | | 1.540167 |
| Elk3 |  | 0.020233 |  | | 1.540147 |
| Olfr153 |  | 0.03998 |  | | 1.539872 |
| Mef2a |  | 0.001494 |  | | 1.539851 |
| 9230110C19Rik |  | 0.012082 |  | | 1.539279 |
| AF067063 |  | 0.001425 |  | | 1.539164 |
| Gm4975 |  | 0.032378 |  | | 1.539138 |
| 4921513D23Rik |  | 0.000239 |  | | 1.538684 |
| Hoxa9 |  | 0.003138 |  | | 1.538628 |
| 6820426E19Rik |  | 0.001522 |  | | 1.538012 |
| Gm16223 |  | 0.014835 |  | | 1.537769 |
| D2Bwg0886e |  | 0.003487 |  | | 1.535116 |
| Samd9l |  | 8.94E-05 |  | | 1.533578 |
| Mc3r |  | 0.00672 |  | | 1.533227 |
| D630033O11Rik |  | 0.005395 |  | | 1.532971 |
| 8430426H19Rik |  | 0.010376 |  | | 1.532765 |
| Atp8a1 |  | 0.002162 |  | | 1.532492 |
| BB094273 |  | 0.010752 |  | | 1.532197 |
| Zfp658 |  | 0.000699 |  | | 1.531619 |
| Slc13a2 |  | 0.016694 |  | | 1.531067 |
| Emp2 |  | 0.002072 |  | | 1.529348 |
| Oas1d |  | 0.022694 |  | | 1.529302 |
| C030039L03Rik |  | 0.000901 |  | | 1.528044 |
| Cma2 |  | 0.008488 |  | | 1.527632 |
| LOC625360 |  | 0.000682 |  | | 1.527498 |
| Itgb3 |  | 0.005562 |  | | 1.527234 |
| Pnpla3 |  | 0.019417 |  | | 1.526909 |
| Hrnr |  | 0.00729 |  | | 1.526838 |
| Nhlh1 |  | 0.009896 |  | | 1.526591 |
| Gm1983 |  | 0.012428 |  | | 1.526525 |
| AI118078 |  | 0.00025 |  | | 1.526519 |
| LOC628205 |  | 0.006004 |  | | 1.526393 |
| Mefv |  | 0.01353 |  | | 1.525968 |
| Slc13a5 |  | 0.000321 |  | | 1.525653 |
| Gm4921 |  | 5.6E-05 |  | | 1.52556 |
| Jdp2 |  | 0.002607 |  | | 1.525153 |
| Msi1 |  | 0.002146 |  | | 1.525135 |
| Egfbp2 |  | 0.03196 |  | | 1.524362 |
| LOC100046488 |  | 0.004826 |  | | 1.523992 |
| 9930111J21Rik1 |  | 0.000968 |  | | 1.523947 |
| Elane |  | 0.007223 |  | | 1.52347 |
| 4833415N18Rik |  | 0.000131 |  | | 1.523349 |
| Ildr1 |  | 0.000871 |  | | 1.523298 |
| Fmn2 |  | 0.014546 |  | | 1.523227 |
| Fam13c |  | 0.022216 |  | | 1.523061 |
| Gm9340 |  | 0.029499 |  | | 1.52297 |
| Cntn1 |  | 0.000692 |  | | 1.522948 |
| Mapt |  | 0.000771 |  | | 1.522872 |
| Gm10404 |  | 0.000281 |  | | 1.522765 |
| Tiam2 |  | 0.000275 |  | | 1.52263 |
| Trp53bp2 |  | 0.002306 |  | | 1.520659 |
| Acox3 |  | 0.000795 |  | | 1.520257 |
| Gm2285 |  | 0.00178 |  | | 1.519971 |
| Ffar3 |  | 0.000426 |  | | 1.519733 |
| Zbp1 |  | 0.00442 |  | | 1.51973 |
| Vpreb2 |  | 0.019316 |  | | 1.51956 |
| Slit2 |  | 0.017259 |  | | 1.518789 |
| Tas2r113 |  | 0.019194 |  | | 1.518721 |
| Gm4884 |  | 0.002342 |  | | 1.518523 |
| Rgs6 |  | 0.003187 |  | | 1.51827 |
| Slc38a11 |  | 0.021931 |  | | 1.517988 |
| Klk1 |  | 0.009968 |  | | 1.517147 |
| Gm3049 |  | 0.002194 |  | | 1.517031 |
| Dub1a |  | 0.001599 |  | | 1.516887 |
| Adra2b |  | 0.000623 |  | | 1.516634 |
| LOC674921 |  | 0.003651 |  | | 1.516588 |
| Lmcd1 |  | 0.00375 |  | | 1.515185 |
| Zfp708 |  | 0.003962 |  | | 1.514897 |
| C87414 |  | 0.041839 |  | | 1.514692 |
| Mog |  | 0.000747 |  | | 1.514437 |
| Scml4 |  | 0.005034 |  | | 1.514374 |
| Tmem92-ps |  | 0.039495 |  | | 1.514187 |
| LOC100046241 |  | 0.000983 |  | | 1.513547 |
| Kng2 |  | 0.00082 |  | | 1.513493 |
| Atp6v0a4 |  | 0.006365 |  | | 1.513336 |
| Gphb5 |  | 0.037046 |  | | 1.512533 |
| Sv2c |  | 0.018165 |  | | 1.512205 |
| Hist1h2aa |  | 0.010744 |  | | 1.512049 |
| 4930504D19Rik |  | 0.029095 |  | | 1.510829 |
| Lrg1 |  | 0.043327 |  | | 1.510411 |
| Csf1 |  | 0.008674 |  | | 1.510304 |
| AI836003 |  | 0.038173 |  | | 1.509652 |
| Abcb4 |  | 0.000119 |  | | 1.509623 |
| LOC100045342 |  | 0.006175 |  | | 1.509185 |
| Itga9 |  | 0.00011 |  | | 1.50917 |
| AU015836 |  | 0.026281 |  | | 1.508726 |
| Spats2l |  | 0.04986 |  | | 1.50783 |
| Gm6835 |  | 0.003224 |  | | 1.50779 |
| Rxfp1 |  | 0.037943 |  | | 1.507633 |
| Cidea |  | 0.001313 |  | | 1.506888 |
| Gng4 |  | 0.009449 |  | | 1.506839 |
| Ccl21a |  | 0.019502 |  | | 1.506109 |
| Gm2640 |  | 0.044938 |  | | 1.505434 |
| Layn |  | 0.01271 |  | | 1.504966 |
| Nr2f2 |  | 0.01711 |  | | 1.504836 |
| LOC636649 |  | 0.00276 |  | | 1.504739 |
| Lepr |  | 0.01552 |  | | 1.504527 |
| Gm8252 |  | 0.025732 |  | | 1.504233 |
| Hhat |  | 0.004641 |  | | 1.503683 |
| 4930546G22Rik |  | 0.020892 |  | | 1.50357 |
| Tgm3 |  | 0.00204 |  | | 1.503158 |
| Spaca4 |  | 0.003073 |  | | 1.503008 |
| LOC100048827 |  | 0.001105 |  | | 1.502416 |
| Gm4733 |  | 0.013726 |  | | 1.502187 |
| Ctnnd2 |  | 0.003054 |  | | 1.502106 |
| AI464131 |  | 1.35E-05 |  | | 1.502042 |
| LOC100044229 |  | 0.008323 |  | | 1.501884 |
| Gm2016 |  | 0.008943 |  | | 1.501867 |
| Hecw2 |  | 0.017944 |  | | 1.501466 |
| Olfr821 |  | 0.010869 |  | | 1.501124 |
| Hist1h2al |  | 0.015567 |  | | 1.501053 |
| Krt80 |  | 0.006428 |  | | 1.501052 |
| Prss29 |  | 0.04093 |  | | 1.500837 |
| Ltc4s |  | 0.032984 |  | | 1.500438 |
| Lamb3 |  | 0.010348 |  | | 1.500108 |
| Klf17 |  | 0.000279 |  | | 0.666582 |
| Gbx2 |  | 0.002035 |  | | 0.666084 |
| 1110054M08Rik |  | 0.016478 |  | | 0.665988 |
| Fam71f2 |  | 0.000908 |  | | 0.66598 |
| Mkrn1 |  | 0.001169 |  | | 0.665903 |
| Agtr1a |  | 0.007578 |  | | 0.665699 |
| Doc2g |  | 0.002189 |  | | 0.665653 |
| 4930524B15Rik |  | 0.008936 |  | | 0.665456 |
| Tmem191c |  | 0.011529 |  | | 0.665445 |
| Tex12 |  | 0.003286 |  | | 0.66514 |
| 4930568B11Rik |  | 0.023088 |  | | 0.664591 |
| LOC640020 |  | 0.002988 |  | | 0.664577 |
| Adk |  | 0.016328 |  | | 0.664035 |
| Spag4 |  | 0.000782 |  | | 0.663772 |
| Flywch2 |  | 5.03E-05 |  | | 0.663587 |
| Prph2 |  | 0.008742 |  | | 0.663514 |
| Jhdm1d |  | 0.022894 |  | | 0.663333 |
| St3gal1 |  | 0.043591 |  | | 0.663265 |
| Lrfn3 |  | 0.002764 |  | | 0.663156 |
| Tmem145 |  | 0.003204 |  | | 0.662827 |
| 4833421G17Rik |  | 0.012187 |  | | 0.662634 |
| Dmrtc1c2 |  | 0.000969 |  | | 0.66245 |
| AI854703 |  | 0.037921 |  | | 0.662115 |
| 4933440N22Rik |  | 0.021694 |  | | 0.661793 |
| D11Wsu173e |  | 0.017618 |  | | 0.661761 |
| Tmem145 |  | 0.00014 |  | | 0.661706 |
| Kcnh6 |  | 0.001691 |  | | 0.661679 |
| LOC100047421 |  | 0.045805 |  | | 0.661596 |
| Ptcra |  | 0.002238 |  | | 0.661468 |
| Tbkbp1 |  | 0.000789 |  | | 0.661326 |
| 4930473A06Rik |  | 0.000677 |  | | 0.661248 |
| Rapgef5 |  | 0.000645 |  | | 0.661202 |
| 4930432F04Rik |  | 0.002182 |  | | 0.661004 |
| Espn |  | 0.009136 |  | | 0.660729 |
| Gm9099 |  | 0.030454 |  | | 0.660584 |
| Slc26a10 |  | 2.52E-05 |  | | 0.660331 |
| LOC100047153 |  | 0.037948 |  | | 0.659806 |
| Rhd |  | 0.000664 |  | | 0.659685 |
| Kif1a |  | 0.035725 |  | | 0.659684 |
| Nudt7 |  | 0.006107 |  | | 0.659579 |
| 9230105E10Rik |  | 0.045987 |  | | 0.659567 |
| Cacna1s |  | 0.01469 |  | | 0.659542 |
| 6430550D23Rik |  | 0.024024 |  | | 0.659505 |
| 4933439C10Rik |  | 0.004719 |  | | 0.659022 |
| Slc16a10 |  | 0.010035 |  | | 0.658832 |
| BC018242 |  | 0.024608 |  | | 0.658582 |
| Tardbp |  | 0.001687 |  | | 0.658496 |
| Tec |  | 0.00207 |  | | 0.658284 |
| Zfr2 |  | 0.006542 |  | | 0.658149 |
| Fitm1 |  | 0.015862 |  | | 0.658024 |
| Fzd2 |  | 0.029948 |  | | 0.657984 |
| Scrt1 |  | 0.019379 |  | | 0.65796 |
| 4930515I15 |  | 0.004631 |  | | 0.657747 |
| Pdyn |  | 0.000425 |  | | 0.657467 |
| Rims4 |  | 0.026915 |  | | 0.657338 |
| 4732440D04Rik |  | 0.025549 |  | | 0.65673 |
| Brsk2 |  | 0.039879 |  | | 0.656398 |
| Nat8l |  | 0.008151 |  | | 0.656315 |
| LOC674503 |  | 0.003896 |  | | 0.656278 |
| Sdccag3 |  | 0.014321 |  | | 0.656085 |
| D330045A20Rik |  | 0.019784 |  | | 0.655737 |
| H2-Q8 |  | 0.001008 |  | | 0.655731 |
| Ctsk |  | 0.026446 |  | | 0.655133 |
| Naa38 |  | 0.000952 |  | | 0.654939 |
| Gm4372 |  | 0.005369 |  | | 0.654767 |
| Tceal6 |  | 0.015628 |  | | 0.654287 |
| Stap2 |  | 0.00385 |  | | 0.654012 |
| Gm15023 |  | 0.021403 |  | | 0.653848 |
| 6030422M02Rik |  | 0.039149 |  | | 0.653756 |
| Catsperg1 |  | 0.025523 |  | | 0.653613 |
| Zfp389 |  | 0.027244 |  | | 0.653567 |
| Lef1 |  | 0.00503 |  | | 0.653376 |
| Zcchc12 |  | 0.000177 |  | | 0.653069 |
| Lpar6 |  | 0.000659 |  | | 0.652752 |
| Tcf15 |  | 0.003221 |  | | 0.652403 |
| Sgce |  | 0.001069 |  | | 0.652158 |
| Hopx |  | 0.00253 |  | | 0.651717 |
| Gata6 |  | 0.004842 |  | | 0.651675 |
| Klc3 |  | 0.008848 |  | | 0.651446 |
| A830010M20Rik |  | 0.006457 |  | | 0.651237 |
| Slc12a9 |  | 0.00957 |  | | 0.650304 |
| Fut10 |  | 0.001649 |  | | 0.650189 |
| Zmym3 |  | 0.01246 |  | | 0.649888 |
| Cldn5 |  | 0.008396 |  | | 0.649599 |
| Htr3a |  | 0.003434 |  | | 0.649582 |
| 1810019N24Rik |  | 0.028693 |  | | 0.649506 |
| Pfdn2 |  | 0.013521 |  | | 0.64913 |
| Iffo2 |  | 0.009185 |  | | 0.64892 |
| Nlrc4 |  | 0.003214 |  | | 0.648301 |
| 1700019B21Rik |  | 0.015582 |  | | 0.648162 |
| Dcc |  | 0.017538 |  | | 0.648128 |
| Zfp606 |  | 0.000604 |  | | 0.647842 |
| Ldhd |  | 0.01754 |  | | 0.647154 |
| Smpdl3a |  | 0.042941 |  | | 0.646502 |
| Zfp369 |  | 0.006373 |  | | 0.646364 |
| 4930471M09Rik |  | 0.001942 |  | | 0.646156 |
| B230369F24Rik |  | 0.004418 |  | | 0.64602 |
| Pim2 |  | 0.010473 |  | | 0.645749 |
| Rilpl1 |  | 0.011279 |  | | 0.645406 |
| Amy1 |  | 0.030616 |  | | 0.645311 |
| Gm7710 |  | 0.003621 |  | | 0.645279 |
| Ttc5 |  | 0.046253 |  | | 0.64524 |
| Eepd1 |  | 0.049512 |  | | 0.645165 |
| Slc1a6 |  | 0.0021 |  | | 0.644081 |
| AI132487 |  | 0.007359 |  | | 0.643815 |
| Lypd3 |  | 0.020962 |  | | 0.643706 |
| Wasf3 |  | 0.004322 |  | | 0.643491 |
| Pdk1 |  | 0.002611 |  | | 0.643354 |
| Stard13 |  | 0.04555 |  | | 0.643325 |
| Mamdc4 |  | 0.007735 |  | | 0.643161 |
| Pogk |  | 0.001342 |  | | 0.642967 |
| F630201L12Rik |  | 0.009307 |  | | 0.642695 |
| Ptpn3 |  | 0.003411 |  | | 0.642535 |
| LOC100045015 |  | 0.03519 |  | | 0.642058 |
| Gm12758 |  | 0.001577 |  | | 0.641683 |
| Stra8 |  | 0.008886 |  | | 0.641512 |
| Rtn2 |  | 0.034807 |  | | 0.640994 |
| Fam102b |  | 0.009279 |  | | 0.640912 |
| Prr22 |  | 0.005386 |  | | 0.640199 |
| Ms4a15 |  | 0.01048 |  | | 0.63996 |
| Ccdc163 |  | 0.02627 |  | | 0.639705 |
| BC005764 |  | 0.000223 |  | | 0.639603 |
| Gm1564 |  | 0.001463 |  | | 0.639109 |
| Nkd1 |  | 0.003205 |  | | 0.638744 |
| A330106F07Rik |  | 0.012986 |  | | 0.638438 |
| Zcwpw1 |  | 0.040632 |  | | 0.637861 |
| Gm6658 |  | 0.000645 |  | | 0.637827 |
| B430105G09Rik |  | 0.034885 |  | | 0.637544 |
| Atp10a |  | 0.001974 |  | | 0.637278 |
| Fam124a |  | 0.037723 |  | | 0.636919 |
| Mapk8ip3 |  | 0.003285 |  | | 0.636211 |
| Dmc1 |  | 0.030878 |  | | 0.636107 |
| Gm4455 |  | 0.046813 |  | | 0.635836 |
| Dbc1 |  | 0.04205 |  | | 0.635834 |
| 4921531C22Rik |  | 0.005522 |  | | 0.635628 |
| Cldn7 |  | 0.03142 |  | | 0.635456 |
| Sdr42e1 |  | 0.021238 |  | | 0.635301 |
| Mtmr1 |  | 0.026934 |  | | 0.63526 |
| Peg10 |  | 0.021451 |  | | 0.635074 |
| 1700001J03Rik |  | 0.031479 |  | | 0.633966 |
| 9530082P21Rik |  | 0.011737 |  | | 0.633268 |
| Zfp677 |  | 0.013342 |  | | 0.632946 |
| 2410012E07Rik |  | 0.000318 |  | | 0.632778 |
| Ceacam16 |  | 0.009744 |  | | 0.632041 |
| Pik3cd |  | 0.027131 |  | | 0.631918 |
| Rgs2 |  | 0.039913 |  | | 0.631797 |
| Antxr2 |  | 0.030396 |  | | 0.631411 |
| Inadl |  | 0.031846 |  | | 0.630839 |
| Pla2g12b |  | 0.013169 |  | | 0.63074 |
| Ahi1 |  | 0.003733 |  | | 0.630507 |
| 2610018G03Rik |  | 0.000393 |  | | 0.630491 |
| Gm9468 |  | 0.040535 |  | | 0.630405 |
| Vsig2 |  | 0.016725 |  | | 0.630383 |
| Gm2485 |  | 0.012891 |  | | 0.630032 |
| Ethe1 |  | 0.026345 |  | | 0.629998 |
| Adra2c |  | 0.02363 |  | | 0.629778 |
| Ccdc88b |  | 0.000909 |  | | 0.629606 |
| Dennd2d |  | 0.005826 |  | | 0.628966 |
| Klra1 |  | 0.013798 |  | | 0.628792 |
| Gnao1 |  | 0.038941 |  | | 0.628351 |
| 4930502E18Rik |  | 0.004189 |  | | 0.628085 |
| Xlr5c |  | 0.016498 |  | | 0.627809 |
| Mir17hg |  | 0.002168 |  | | 0.627623 |
| 5830416P10Rik |  | 0.034922 |  | | 0.627254 |
| Hnrnpa0 |  | 0.001877 |  | | 0.627203 |
| Chrna5 |  | 0.035019 |  | | 0.626265 |
| Fbf1 |  | 0.018784 |  | | 0.625966 |
| Pld4 |  | 0.032702 |  | | 0.625723 |
| 6720456H20Rik |  | 0.001563 |  | | 0.624879 |
| Nme5 |  | 0.015401 |  | | 0.624726 |
| Etv1 |  | 0.0029 |  | | 0.624414 |
| Stbd1 |  | 0.002187 |  | | 0.6241 |
| Gm5124 |  | 0.001126 |  | | 0.623873 |
| Pex6 |  | 0.001937 |  | | 0.623407 |
| Slc35f3 |  | 0.002624 |  | | 0.622825 |
| Myh3 |  | 0.001297 |  | | 0.622817 |
| Trfr2 |  | 0.000792 |  | | 0.622516 |
| LOC100303645 |  | 0.000841 |  | | 0.622233 |
| Gm3876 |  | 0.012962 |  | | 0.622093 |
| Slc38a3 |  | 0.00037 |  | | 0.622006 |
| 5430405G05Rik |  | 0.018835 |  | | 0.621394 |
| Thbs4 |  | 0.016115 |  | | 0.62124 |
| Cgnl1 |  | 0.007054 |  | | 0.620876 |
| Gm3237 |  | 0.03138 |  | | 0.620105 |
| Wdr54 |  | 0.042075 |  | | 0.619469 |
| Sel1l3 |  | 0.004903 |  | | 0.6194 |
| Rnf43 |  | 0.024854 |  | | 0.619353 |
| 9030625N01Rik |  | 0.014209 |  | | 0.619142 |
| Zfp467 |  | 0.016506 |  | | 0.618597 |
| 3110027N22Rik |  | 0.017716 |  | | 0.618312 |
| Inca1 |  | 0.000637 |  | | 0.61785 |
| Tktl2 |  | 0.004748 |  | | 0.617052 |
| AY074887 |  | 0.003418 |  | | 0.616891 |
| Paqr6 |  | 0.015042 |  | | 0.61662 |
| Vipr1 |  | 0.037465 |  | | 0.616067 |
| LOC630474 |  | 0.034121 |  | | 0.616018 |
| LOC100047647 |  | 0.044503 |  | | 0.615805 |
| Pim1 |  | 0.003192 |  | | 0.615162 |
| Hcst |  | 0.003117 |  | | 0.615062 |
| Cass4 |  | 0.03666 |  | | 0.615054 |
| Cd164l2 |  | 0.021476 |  | | 0.614639 |
| Mogat2 |  | 0.01273 |  | | 0.613906 |
| 2610528J11Rik |  | 0.042745 |  | | 0.613074 |
| LOC100047189 |  | 0.018471 |  | | 0.612166 |
| 2900024J01Rik |  | 0.017137 |  | | 0.611685 |
| 6530418L21Rik |  | 0.023364 |  | | 0.611665 |
| 7420416P09Rik |  | 0.001474 |  | | 0.61154 |
| Gypc |  | 0.004551 |  | | 0.611471 |
| Gm1564 |  | 0.006226 |  | | 0.610504 |
| Myh3 |  | 0.000112 |  | | 0.609912 |
| A930033H14Rik |  | 0.012016 |  | | 0.609869 |
| Meg3 |  | 3.48E-06 |  | | 0.6097 |
| 2900006K08Rik |  | 0.01676 |  | | 0.609023 |
| 1700011F14Rik |  | 0.001765 |  | | 0.608759 |
| B630005N14Rik |  | 0.026737 |  | | 0.608684 |
| Gm13033 |  | 0.011688 |  | | 0.608515 |
| Fndc3c1 |  | 0.014082 |  | | 0.60798 |
| Cubn |  | 0.007379 |  | | 0.607807 |
| Asphd1 |  | 0.007241 |  | | 0.607563 |
| Sbsn |  | 0.000488 |  | | 0.607456 |
| Wfdc10 |  | 0.006361 |  | | 0.606796 |
| Gpm6b |  | 0.000707 |  | | 0.606451 |
| Cpne1 |  | 0.008008 |  | | 0.606198 |
| Tmem176b |  | 0.003549 |  | | 0.606157 |
| Gm1006 |  | 0.000261 |  | | 0.605689 |
| Atf7 |  | 0.015734 |  | | 0.605443 |
| Igsf1 |  | 0.03179 |  | | 0.605073 |
| Moxd1 |  | 0.018028 |  | | 0.605018 |
| Gm6310 |  | 0.002817 |  | | 0.604535 |
| Amn |  | 0.008603 |  | | 0.604457 |
| S100z |  | 0.001588 |  | | 0.604142 |
| 5031434O11Rik |  | 0.011292 |  | | 0.604083 |
| 6430598A04Rik |  | 0.010567 |  | | 0.60408 |
| Ssxb10 |  | 0.009337 |  | | 0.602844 |
| C130045F17Rik |  | 0.007748 |  | | 0.601889 |
| C030034I22Rik |  | 0.006815 |  | | 0.601813 |
| 5430411C19Rik |  | 0.001777 |  | | 0.600948 |
| Dydc2 |  | 0.047926 |  | | 0.60018 |
| Hdx |  | 0.009634 |  | | 0.599864 |
| Cthrc1 |  | 0.000819 |  | | 0.59954 |
| Acer2 |  | 0.003679 |  | | 0.599454 |
| Ano10 |  | 0.002929 |  | | 0.599222 |
| Vsig10 |  | 0.046124 |  | | 0.598618 |
| Slc16a2 |  | 0.012482 |  | | 0.598579 |
| Snhg11 |  | 0.006331 |  | | 0.598383 |
| Bai2 |  | 0.005109 |  | | 0.597728 |
| Gm2943 |  | 0.005367 |  | | 0.597333 |
| Sema6a |  | 0.003453 |  | | 0.596228 |
| A430105J06Rik |  | 0.040333 |  | | 0.595833 |
| BC050777 |  | 0.022912 |  | | 0.595562 |
| Nhlrc4 |  | 0.010468 |  | | 0.595534 |
| Rgs17 |  | 0.048748 |  | | 0.595502 |
| 4930423D22Rik |  | 0.031031 |  | | 0.595297 |
| Rasgef1b |  | 0.000338 |  | | 0.594824 |
| Hpn |  | 0.001055 |  | | 0.594502 |
| LOC546296 |  | 0.005694 |  | | 0.594454 |
| Gm4132 |  | 0.02004 |  | | 0.594384 |
| Wt1 |  | 0.000903 |  | | 0.593831 |
| B630019K06Rik |  | 0.011673 |  | | 0.593176 |
| Gm44 |  | 0.000166 |  | | 0.593102 |
| 1700001G17Rik |  | 0.025772 |  | | 0.592903 |
| Prmt6 |  | 0.009261 |  | | 0.591752 |
| Fam84a |  | 0.009044 |  | | 0.591096 |
| Map3k10 |  | 0.016504 |  | | 0.590968 |
| Ppm1j |  | 0.000233 |  | | 0.590129 |
| Epha7 |  | 0.000364 |  | | 0.589731 |
| Epha8 |  | 0.000371 |  | | 0.589385 |
| Zyx |  | 0.007168 |  | | 0.587915 |
| Gm6306 |  | 2.95E-05 |  | | 0.587644 |
| LOC100045152 |  | 0.005186 |  | | 0.587142 |
| Crlf1 |  | 0.000391 |  | | 0.58637 |
| Tex13 |  | 8.51E-05 |  | | 0.58582 |
| LOC100046773 |  | 0.041164 |  | | 0.584677 |
| 4930432N10Rik |  | 0.041646 |  | | 0.584279 |
| Gm4480 |  | 0.016614 |  | | 0.58391 |
| Irf8 |  | 0.036501 |  | | 0.583305 |
| Trf |  | 0.002511 |  | | 0.583238 |
| Eef2k |  | 0.010141 |  | | 0.581851 |
| Bcl6b |  | 0.000125 |  | | 0.581415 |
| E530001K10Rik |  | 0.003611 |  | | 0.581189 |
| Katnal2 |  | 0.024496 |  | | 0.58115 |
| Gm2483 |  | 2.92E-05 |  | | 0.580927 |
| Hist1h2ac |  | 0.000476 |  | | 0.580787 |
| Zar1l |  | 0.043879 |  | | 0.580657 |
| Myc |  | 0.000445 |  | | 0.58009 |
| LOC100047469 |  | 0.001006 |  | | 0.579953 |
| A430104N18Rik |  | 0.013103 |  | | 0.579611 |
| Emid1 |  | 0.022588 |  | | 0.579084 |
| E330037I15Rik |  | 0.006871 |  | | 0.578919 |
| Bmp6 |  | 0.020224 |  | | 0.578906 |
| AI662501 |  | 0.002187 |  | | 0.578765 |
| Mertk |  | 9.29E-05 |  | | 0.578673 |
| Bcl11b |  | 0.007288 |  | | 0.578587 |
| Ccdc79 |  | 0.020223 |  | | 0.577394 |
| Gm2701 |  | 0.010789 |  | | 0.57708 |
| LOC100045113 |  | 0.036085 |  | | 0.57699 |
| LOC100046931 |  | 0.009169 |  | | 0.576189 |
| Gna14 |  | 0.002777 |  | | 0.575724 |
| 5430405G05Rik |  | 0.028556 |  | | 0.575193 |
| Gm98 |  | 0.047201 |  | | 0.573566 |
| Zfp768 |  | 0.00094 |  | | 0.572123 |
| C430049E01Rik |  | 0.044574 |  | | 0.571366 |
| Cyth4 |  | 0.003115 |  | | 0.571064 |
| Apob48r |  | 0.004276 |  | | 0.570577 |
| Dnajb2 |  | 0.022077 |  | | 0.570402 |
| Slc13a3 |  | 0.007521 |  | | 0.569072 |
| Ano10 |  | 0.021527 |  | | 0.568904 |
| Eid3 |  | 0.013188 |  | | 0.568512 |
| 4932413F04Rik |  | 0.047649 |  | | 0.567957 |
| Vgll4 |  | 0.042346 |  | | 0.567871 |
| Tmem114 |  | 0.037707 |  | | 0.567669 |
| Fam19a4 |  | 0.00046 |  | | 0.567363 |
| Trf |  | 2.82E-05 |  | | 0.567243 |
| Olfr1383 |  | 0.016811 |  | | 0.567152 |
| 1700048O20Rik |  | 0.036516 |  | | 0.566995 |
| LOC633721 |  | 0.045359 |  | | 0.566796 |
| Kirrel2 |  | 0.004846 |  | | 0.56533 |
| Katnal1 |  | 0.002097 |  | | 0.564303 |
| Aqp8 |  | 0.00381 |  | | 0.56407 |
| LOC100048747 |  | 0.00131 |  | | 0.563986 |
| Gm6556 |  | 0.003425 |  | | 0.563778 |
| 9430078G10Rik |  | 0.036994 |  | | 0.562368 |
| Gm4123 |  | 0.001165 |  | | 0.558872 |
| Abi3 |  | 0.030294 |  | | 0.558619 |
| Gm5468 |  | 0.040543 |  | | 0.557879 |
| C230076A16Rik |  | 0.008841 |  | | 0.557849 |
| Ttc21b |  | 0.00945 |  | | 0.557742 |
| 2610008G14Rik |  | 0.012547 |  | | 0.557672 |
| LOC100047698 |  | 0.022141 |  | | 0.557402 |
| Ggnbp1 |  | 0.001735 |  | | 0.556921 |
| LOC100048601 |  | 0.002335 |  | | 0.556429 |
| Txnip |  | 0.00024 |  | | 0.555891 |
| Hoxb4 |  | 0.005989 |  | | 0.555878 |
| Pcdh8 |  | 0.018768 |  | | 0.55559 |
| Khdrbs1 |  | 0.000643 |  | | 0.555364 |
| Clip2 |  | 0.004253 |  | | 0.555131 |
| Sez6l2 |  | 0.030392 |  | | 0.55255 |
| Bnipl |  | 0.017237 |  | | 0.551743 |
| B930095G15Rik |  | 0.045363 |  | | 0.55154 |
| Col6a4 |  | 0.040082 |  | | 0.551147 |
| A630026N12Rik |  | 0.016021 |  | | 0.551079 |
| Jph4 |  | 0.000439 |  | | 0.551021 |
| Fam19a4 |  | 0.028039 |  | | 0.550908 |
| Hoxb2 |  | 0.002655 |  | | 0.550107 |
| Mdfi |  | 0.011105 |  | | 0.549992 |
| 6430573F11Rik |  | 0.018991 |  | | 0.549107 |
| 2810008D09Rik |  | 0.001957 |  | | 0.548836 |
| Uprt |  | 0.033253 |  | | 0.548797 |
| Gm2238 |  | 0.034548 |  | | 0.548335 |
| Stac3 |  | 0.021688 |  | | 0.548111 |
| Zfp787 |  | 0.038562 |  | | 0.548095 |
| Rnf122 |  | 0.045297 |  | | 0.547222 |
| Gm7455 |  | 0.000795 |  | | 0.546859 |
| Hoxb13 |  | 3.12E-05 |  | | 0.546692 |
| Gm13124 |  | 0.008388 |  | | 0.546446 |
| Akr1b7 |  | 0.022714 |  | | 0.54522 |
| Treml2 |  | 0.002875 |  | | 0.545107 |
| Cela1 |  | 0.020571 |  | | 0.544539 |
| Sh2b2 |  | 0.002307 |  | | 0.544217 |
| Cyp11a1 |  | 0.002804 |  | | 0.541807 |
| Tenc1 |  | 0.007736 |  | | 0.540284 |
| Comp |  | 0.008274 |  | | 0.540235 |
| Cdc42bpg |  | 0.007232 |  | | 0.539816 |
| 1700123K08Rik |  | 0.001889 |  | | 0.538899 |
| Gm5464 |  | 0.001253 |  | | 0.53642 |
| Clca3 |  | 0.000789 |  | | 0.536131 |
| Hoxb7 |  | 0.000851 |  | | 0.53612 |
| Gprin1 |  | 0.000897 |  | | 0.536007 |
| Zfp558-ps |  | 0.025562 |  | | 0.535964 |
| 9430024F10Rik |  | 0.010781 |  | | 0.535155 |
| Cbx6-Nptxr |  | 0.003939 |  | | 0.535124 |
| LOC100047123 |  | 0.049269 |  | | 0.533388 |
| 6820445E23Rik |  | 0.011094 |  | | 0.533351 |
| Thbs2 |  | 0.001536 |  | | 0.53249 |
| Ctsh |  | 0.005335 |  | | 0.53126 |
| AI480461 |  | 0.043234 |  | | 0.529817 |
| Malat1 |  | 0.04166 |  | | 0.529455 |
| Tceal3 |  | 0.019061 |  | | 0.52739 |
| Meg3 |  | 0.007005 |  | | 0.527193 |
| Slc39a8 |  | 0.00723 |  | | 0.527104 |
| Tmem221 |  | 0.004218 |  | | 0.52655 |
| Dusp6 |  | 5.58E-05 |  | | 0.525623 |
| 4930579G22Rik |  | 0.004137 |  | | 0.524158 |
| Pik3r5 |  | 0.004097 |  | | 0.524066 |
| 2810405F17Rik |  | 0.003486 |  | | 0.521525 |
| Tal2 |  | 0.001823 |  | | 0.520551 |
| Txnip |  | 0.000507 |  | | 0.519366 |
| Fcgbp |  | 0.004546 |  | | 0.519276 |
| 9430069I07Rik |  | 0.000172 |  | | 0.519061 |
| Cnr1 |  | 0.001418 |  | | 0.518986 |
| Hoxb5 |  | 0.006367 |  | | 0.517486 |
| Snora74a |  | 7.05E-05 |  | | 0.517303 |
| AY074887 |  | 0.004709 |  | | 0.514938 |
| LOC100046427 |  | 0.000337 |  | | 0.512593 |
| Naprt1 |  | 0.000395 |  | | 0.511849 |
| 5033425G24Rik |  | 0.020332 |  | | 0.510668 |
| Armcx6 |  | 0.025404 |  | | 0.508902 |
| Fut4 |  | 0.028891 |  | | 0.507667 |
| Prdm9 |  | 0.036034 |  | | 0.505784 |
| Cstad |  | 0.022843 |  | | 0.50543 |
| Cdkn1a |  | 0.026323 |  | | 0.502816 |
| Gm10638 |  | 0.007201 |  | | 0.50269 |
| 1700011F14Rik |  | 0.00771 |  | | 0.50228 |
| Cdh10 |  | 0.005855 |  | | 0.500477 |
| Gm5423 |  | 0.032184 |  | | 0.500012 |
| Etv4 |  | 0.001405 |  | | 0.498333 |
| Atp4a |  | 0.021027 |  | | 0.497249 |
| Msh4 |  | 0.010725 |  | | 0.496194 |
| 4933435E02Rik |  | 0.009721 |  | | 0.494554 |
| Wdr38 |  | 0.014834 |  | | 0.493966 |
| Robo3 |  | 0.015934 |  | | 0.49308 |
| Wbscr25 |  | 0.003553 |  | | 0.492482 |
| 9330198I05Rik |  | 0.03468 |  | | 0.49122 |
| Prss44 |  | 0.010801 |  | | 0.489457 |
| 2510016G02Rik |  | 0.000715 |  | | 0.487914 |
| Mmd2 |  | 0.005832 |  | | 0.487476 |
| Gm2509 |  | 0.023324 |  | | 0.485272 |
| Cntfr |  | 0.008258 |  | | 0.483012 |
| Rgs20 |  | 0.008395 |  | | 0.479768 |
| Il25 |  | 0.009487 |  | | 0.478818 |
| 5031415H12Rik |  | 0.000177 |  | | 0.477514 |
| Hs6st2 |  | 0.005346 |  | | 0.475959 |
| Dusp4 |  | 0.00029 |  | | 0.473482 |
| Etv4 |  | 0.006719 |  | | 0.464797 |
| Triobp |  | 0.003459 |  | | 0.463043 |
| Mbp |  | 0.026591 |  | | 0.461917 |
| Gm11538 |  | 1.4E-05 |  | | 0.449122 |
| Lgals2 |  | 0.002416 |  | | 0.442061 |
| Tcf23 |  | 0.001003 |  | | 0.438942 |
| 4933417C20Rik |  | 0.006464 |  | | 0.437001 |
| Dgkb |  | 0.011705 |  | | 0.43381 |
| Rxrg |  | 0.001948 |  | | 0.423382 |
| Wnt8a |  | 0.000291 |  | | 0.420801 |
| 2400009B08Rik |  | 0.014673 |  | | 0.412767 |
| Gm10766 |  | 0.007613 |  | | 0.406167 |
| 4930465K10Rik |  | 0.029142 |  | | 0.39216 |
| Gm5091 |  | 0.006521 |  | | 0.38785 |
| Gcm1 |  | 0.004241 |  | | 0.386495 |
| Hoxb1 |  | 0.004517 |  | | 0.377467 |
| 4921532D01Rik |  | 0.018053 |  | | 0.374946 |
| Gm10050 |  | 0.001382 |  | | 0.342672 |
| Epha1 |  | 0.002159 |  | | 0.271337 |
| Egr1 |  | 3.18E-05 |  | | 0.037873 |

**Supplementary Table S2. Differentially expressed miRNAs in PD0325901 (PD) treated J1 ES cells. Fold change (FC) values are provided in comparison with the control ESCs which were maintained in standard ES cells medium without the addition of PD0325901.**

| miR-name |  | FC | | p-value |
| --- | --- | --- | --- | --- |
| mmu-miR-871-3p |  | 4.143773595 | 0.001449056 | |
| mmu-miR-331-3p |  | 3.638486884 | 9.43E-49 | |
| mmu-miR-188-5p |  | 2.432723155 | 0.01300827 | |
| mmu-miR-302d-3p |  | 2.330721587 | 0.000000337 | |
| mmu-miR-302d-5p |  | 2.302579151 | 0.029785469 | |
| mmu-miR-21-3p |  | 2.202558183 | 0.0420854 | |
| mmu-miR-328-3p |  | 2.202558183 | 0.0420854 | |
| mmu-miR-3471 |  | 2.12969634 | 0.032160254 | |
| mmu-miR-504-5p |  | 2.027125838 | 0.016353651 | |
| mmu-miR-3096b-5p |  | 1.944372763 | 4.02E-18 | |
| mmu-miR-1194 |  | 1.891970941 | 0.016994039 | |
| mmu-miR-669f-3p |  | 1.887680838 | 0.01480142 | |
| mmu-miR-124-3p |  | 1.849625509 | 8.05E-284 | |
| mmu-miR-669a-5p |  | 1.843814451 | 0.00000186 | |
| mmu-miR-669p-5p |  | 1.843814451 | 0.00000186 | |
| mmu-miR-302a-5p |  | 1.75977179 | 7.8E-10 | |
| mmu-miR-339-5p |  | 1.757594512 | 0.00026008 | |
| mmu-miR-467a-5p |  | 1.749130815 | 4.39E-25 | |
| mmu-miR-467b-5p |  | 1.749130815 | 4.39E-25 | |
| mmu-miR-181d-3p |  | 1.676748092 | 0.011332703 | |
| mmu-miR-669o-5p |  | 1.656561965 | 0.022342709 | |
| mmu-miR-3096-5p |  | 1.650429176 | 0.0000132 | |
| mmu-miR-124-5p |  | 1.634309033 | 0.008880721 | |
| mmu-miR-669d-5p |  | 1.628683405 | 0.044556969 | |
| mmu-miR-365-2-5p |  | 1.571052628 | 1.56E-14 | |
| mmu-miR-467d-5p |  | 1.568152842 | 0.000000749 | |
| mmu-miR-465b-5p |  | 0.662448662 | 0.117002763 | |
| mmu-miR-411-5p |  | 0.658228459 | 3.5E-54 | |
| mmu-miR-154-3p |  | 0.655330567 | 9.17E-17 | |
| mmu-miR-3086-5p |  | 0.655201988 | 0.197812046 | |
| mmu-miR-674-3p |  | 0.653700996 | 1.51E-10 | |
| mmu-miR-30c-1-3p |  | 0.652664891 | 0.001707326 | |
| mmu-miR-370-3p |  | 0.652180649 | 0.001249487 | |
| mmu-miR-494-3p |  | 0.652015145 | 6.83E-52 | |
| mmu-miR-5113 |  | 0.643543895 | 0.191867365 | |
| mmu-miR-363-3p |  | 0.640297526 | 1.09E-52 | |
| mmu-miR-148b-3p |  | 0.63840913 | 1.02E-28 | |
| mmu-miR-212-5p |  | 0.635975137 | 0.088258976 | |
| mmu-miR-323-3p |  | 0.635320849 | 0 | |
| mmu-miR-669l-3p |  | 0.630625529 | 0.185403066 | |
| mmu-miR-380-5p |  | 0.629592579 | 0.006460787 | |
| mmu-miR-409-3p |  | 0.627580521 | 1.01E-75 | |
| mmu-miR-344-3p |  | 0.6251396 | 0.034197117 | |
| mmu-miR-433-5p |  | 0.62388081 | 2.73E-11 | |
| mmu-miR-150-5p |  | 0.622552116 | 0.00000306 | |
| mmu-miR-1195 |  | 0.621863045 | 0.0000325 | |
| mmu-miR-434-5p |  | 0.619848436 | 0.000574038 | |
| mmu-miR-136-3p |  | 0.61922441 | 6.26E-08 | |
| mmu-miR-181c-3p |  | 0.614229083 | 2.29E-16 | |
| mmu-miR-539-5p |  | 0.612374872 | 0.00036726 | |
| mmu-miR-10a-5p |  | 0.610130341 | 1.14E-24 | |
| mmu-miR-101b-3p |  | 0.607272418 | 0 | |
| mmu-miR-186-3p |  | 0.607170692 | 0.030559619 | |
| mmu-miR-27b-3p |  | 0.606986389 | 2.27E-104 | |
| mmu-miR-27a-3p |  | 0.606449975 | 4.44E-84 | |
| mmu-miR-149-3p |  | 0.605443719 | 0.000000371 | |
| mmu-miR-677-5p |  | 0.603826233 | 0.001761408 | |
| mmu-miR-541-3p |  | 0.600642256 | 0.014095844 | |
| mmu-miR-5126 |  | 0.600621602 | 0.020279682 | |
| mmu-miR-362-5p |  | 0.592031486 | 0.04922993 | |
| mmu-miR-377-5p |  | 0.589716669 | 2.47E-08 | |
| mmu-miR-1188-5p |  | 0.582821595 | 1.13E-10 | |
| mmu-miR-92a-3p |  | 0.582307348 | 0 | |
| mmu-miR-669m-3p |  | 0.577974793 | 0.011719888 | |
| mmu-miR-129-1-3p |  | 0.573336002 | 0.00436754 | |
| mmu-miR-1943-5p |  | 0.569992681 | 0.013306624 | |
| mmu-miR-181a-2-3p | | 0.568472841 | 0.001113517 | |
| mmu-miR-449a-5p |  | 0.567281708 | 0.001321248 | |
| mmu-miR-1949 |  | 0.564077317 | 0.000112462 | |
| mmu-miR-5105 |  | 0.552158349 | 4.03E-67 | |
| mmu-miR-3079-5p |  | 0.548408269 | 0.074111008 | |
| mmu-miR-16-2-3p |  | 0.540551762 | 0.006930063 | |
| mmu-miR-361-3p |  | 0.540522692 | 0.089793 | |
| mmu-miR-382-3p |  | 0.533721776 | 1.2E-200 | |
| mmu-miR-382-5p |  | 0.533350962 | 0 | |
| mmu-miR-1930-5p |  | 0.51484926 | 0.008387894 | |
| mmu-miR-221-5p |  | 0.500524166 | 0.011617516 | |
| mmu-miR-667-3p |  | 0.498877141 | 0.00000305 | |
| mmu-miR-540-3p |  | 0.493381334 | 6.19E-29 | |
| mmu-miR-466i-3p |  | 0.473167618 | 0.000000107 | |
| mmu-miR-140-5p |  | 0.450500799 | 0.016437415 | |
| mmu-miR-130a-5p |  | 0.440474694 | 0.00000426 | |
| mmu-miR-222-3p |  | 0.416571412 | 1.28E-65 | |
| mmu-miR-126-3p |  | 0.412958013 | 0.012489758 | |
| mmu-miR-5100 |  | 0.412133137 | 0.000000579 | |
| mmu-miR-410-5p |  | 0.401630119 | 1.04E-11 | |
| mmu-miR-206-3p |  | 0.381756541 | 0.0000253 | |
| mmu-miR-1938 |  | 0.245711346 | 0.0000423 | |
| mmu-miR-296-3p |  | 0.232350052 | 2.2223e-315 | |

**Supplementary Table S3. Differentially expressed miRNAs in CHIR99021 (CHIR) treated J1 ES cells. Fold change (FC) values are provided in comparison with the control ESCs which were maintained in standard ES cells medium without the addition of CHIR99021.**

| miR-name |  | fold change | p-value |
| --- | --- | --- | --- |
| mmu-miR-211-3p |  | 51.62075474 | 1.93E-30 |
| mmu-miR-122-5p |  | 27.32282308 | 0 |
| mmu-miR-211-5p |  | 18.15229116 | 6.19E-32 |
| mmu-miR-147-3p |  | 2.722911042 | 0.014712 |
| mmu-miR-10b-5p |  | 2.118002262 | 0.010792 |
| mmu-miR-3471 |  | 2.021787611 | 0.044699 |
| mmu-miR-331-3p |  | 1.991769611 | 4.95E-12 |
| mmu-miR-140-3p |  | 0.662194021 | 0 |
| mmu-miR-106a-5p |  | 0.660075996 | 1.09E-11 |
| mmu-miR-101a-3p |  | 0.649432064 | 0 |
| mmu-miR-872-5p |  | 0.648491927 | 2.37E-25 |
| mmu-miR-143-5p |  | 0.645343147 | 0.000196 |
| mmu-miR-181d-5p |  | 0.643818304 | 0 |
| mmu-miR-291a-3p |  | 0.643381403 | 2.8E-175 |
| mmu-miR-1949 |  | 0.642315644 | 0.001433 |
| mmu-miR-302d-3p |  | 0.638839888 | 0.041395 |
| mmu-miR-23b-5p |  | 0.634022568 | 2.63E-20 |
| mmu-miR-302a-5p |  | 0.631695688 | 5.9E-05 |
| mmu-miR-429-3p |  | 0.631520937 | 2.75E-08 |
| mmu-miR-93-5p |  | 0.628947852 | 1.1E-150 |
| mmu-miR-15a-5p |  | 0.627048426 | 6.76E-13 |
| mmu-miR-652-3p |  | 0.626471759 | 0.008524 |
| mmu-miR-340-5p |  | 0.626194564 | 1.3E-99 |
| mmu-miR-24-3p |  | 0.625418968 | 3.87E-74 |
| mmu-miR-193-3p |  | 0.622306845 | 4.85E-10 |
| mmu-miR-421-3p |  | 0.621596597 | 5.86E-05 |
| mmu-miR-374-5p |  | 0.62111365 | 1.34E-12 |
| mmu-miR-107-3p |  | 0.613931585 | 0 |
| mmu-miR-294-3p |  | 0.613241871 | 0 |
| mmu-miR-29a-3p |  | 0.613188778 | 6.6E-134 |
| mmu-miR-467c-5p |  | 0.611907535 | 5.06E-06 |
| mmu-miR-124-5p |  | 0.609558082 | 0.031351 |
| mmu-miR-450b-3p |  | 0.607375433 | 4.41E-05 |
| mmu-miR-106b-5p |  | 0.60692865 | 4.27E-59 |
| mmu-miR-182-5p |  | 0.603829481 | 0 |
| mmu-miR-16-5p |  | 0.603291412 | 7.5E-131 |
| mmu-miR-3078-5p |  | 0.601060846 | 0.04075 |
| mmu-miR-361-5p |  | 0.600946515 | 3.75E-06 |
| mmu-miR-5128 |  | 0.600151529 | 1.47E-11 |
| mmu-miR-29b-3p |  | 0.599587441 | 1.41E-06 |
| mmu-miR-378-3p |  | 0.597089569 | 1.8E-127 |
| mmu-miR-185-3p |  | 0.595700229 | 0.00175 |
| mmu-miR-1964-3p |  | 0.595659923 | 0.040458 |
| mmu-miR-183-3p |  | 0.593878113 | 2.25E-21 |
| mmu-miR-3474 |  | 0.591480307 | 0.017835 |
| mmu-miR-505-5p |  | 0.589372532 | 7.31E-10 |
| mmu-miR-301a-5p |  | 0.58856811 | 9.76E-33 |
| mmu-miR-16-2-3p |  | 0.587726914 | 0.013555 |
| mmu-miR-1929-5p |  | 0.587726914 | 0.013555 |
| mmu-miR-34b-5p |  | 0.586114003 | 2.4E-05 |
| mmu-miR-20b-5p |  | 0.585813088 | 1.67E-25 |
| mmu-miR-1935 |  | 0.5827964 | 1.22E-31 |
| mmu-miR-669c-5p |  | 0.581812267 | 0 |
| mmu-miR-542-5p |  | 0.580418058 | 0.010197 |
| mmu-miR-872-3p |  | 0.579721931 | 2.74E-11 |
| mmu-miR-155-5p |  | 0.579588924 | 0.02987 |
| mmu-miR-5105 |  | 0.57787858 | 1.7E-62 |
| mmu-miR-363-5p |  | 0.575932252 | 0 |
| mmu-miR-181c-5p |  | 0.573717957 | 8.99E-77 |
| mmu-miR-106b-3p |  | 0.572995852 | 1.19E-71 |
| mmu-miR-23b-3p |  | 0.572952614 | 4.92E-71 |
| mmu-miR-335-5p |  | 0.572129544 | 5.72E-23 |
| mmu-miR-28-5p |  | 0.567333614 | 0.003393 |
| mmu-miR-183-5p |  | 0.566833602 | 4.5E-104 |
| mmu-miR-30e-3p |  | 0.565285392 | 3.18E-08 |
| mmu-miR-34a-3p |  | 0.564150046 | 0.000118 |
| mmu-miR-103-3p |  | 0.562043824 | 0 |
| mmu-miR-1306-3p |  | 0.560994266 | 2.49E-14 |
| mmu-miR-151-3p |  | 0.559728417 | 1.05E-26 |
| mmu-miR-30c-2-3p |  | 0.559656437 | 4.11E-19 |
| mmu-miR-17-5p |  | 0.557509599 | 3.34E-70 |
| mmu-miR-200c-3p |  | 0.557411325 | 7.61E-14 |
| mmu-miR-221-3p |  | 0.557394167 | 1.89E-26 |
| mmu-miR-320-3p |  | 0.556189425 | 0 |
| mmu-miR-466d-3p |  | 0.555981884 | 0.000308 |
| mmu-miR-301a-3p |  | 0.554131744 | 1.41E-05 |
| mmu-miR-362-3p |  | 0.552198839 | 8.25E-05 |
| mmu-miR-3066-5p |  | 0.550640641 | 2.64E-09 |
| mmu-miR-669d-5p |  | 0.549862098 | 0.048979 |
| mmu-miR-292-3p |  | 0.54923958 | 0 |
| mmu-miR-1934-3p |  | 0.548416807 | 4.91E-07 |
| mmu-miR-16-1-3p |  | 0.546975493 | 9.53E-18 |
| mmu-miR-362-5p |  | 0.54464093 | 0.020558 |
| mmu-miR-669h-5p |  | 0.544325678 | 2.74E-06 |
| mmu-miR-540-5p |  | 0.543785331 | 1.08E-09 |
| mmu-miR-148a-3p |  | 0.543761759 | 1.31E-23 |
| mmu-miR-200b-5p |  | 0.541722715 | 3.52E-08 |
| mmu-miR-96-5p |  | 0.541541881 | 3.72E-81 |
| mmu-miR-298-5p |  | 0.539405213 | 0 |
| mmu-miR-3107-5p |  | 0.538954038 | 0.035654 |
| mmu-miR-30a-5p |  | 0.537089715 | 9.3E-149 |
| mmu-miR-301b-3p |  | 0.536482817 | 1.94E-05 |
| mmu-miR-18b-3p |  | 0.536113601 | 0.011394 |
| mmu-miR-21-5p |  | 0.535350083 | 0 |
| mmu-miR-99b-5p |  | 0.532160248 | 2.3E-222 |
| mmu-miR-30c-1-3p |  | 0.531596857 | 5E-06 |
| mmu-miR-302b-3p |  | 0.529493988 | 0.034664 |
| mmu-miR-29c-3p |  | 0.528968435 | 1.59E-19 |
| mmu-miR-290-3p |  | 0.527961623 | 3.65E-14 |
| mmu-miR-15b-5p |  | 0.525212919 | 8.99E-59 |
| mmu-miR-676-3p |  | 0.524863199 | 1.82E-52 |
| mmu-miR-130a-3p |  | 0.524726905 | 0 |
| mmu-miR-30e-5p |  | 0.520009195 | 1.45E-52 |
| mmu-miR-412-3p |  | 0.519306141 | 0.033569 |
| mmu-miR-30b-5p |  | 0.518908038 | 0.000296 |
| mmu-miR-425-3p |  | 0.517777594 | 1.36E-07 |
| mmu-miR-20a-5p |  | 0.517140705 | 3.67E-42 |
| mmu-miR-92a-2-5p |  | 0.515415054 | 4.2E-207 |
| mmu-miR-151-5p |  | 0.5154024 | 1.01E-17 |
| mmu-miR-19b-3p |  | 0.514942585 | 7.56E-38 |
| mmu-miR-342-5p |  | 0.513333618 | 7.6E-14 |
| mmu-miR-466e-5p |  | 0.511846353 | 0.004366 |
| mmu-miR-1968-5p |  | 0.510779143 | 5.32E-14 |
| mmu-miR-30a-3p |  | 0.510479432 | 4.39E-21 |
| mmu-miR-1839-5p |  | 0.510088977 | 0 |
| mmu-miR-99b-3p |  | 0.507823323 | 8.47E-30 |
| mmu-miR-22-5p |  | 0.505446672 | 5.08E-05 |
| mmu-let-7b-5p |  | 0.503665986 | 4.09E-72 |
| mmu-miR-17-3p |  | 0.490641282 | 2.76E-76 |
| mmu-miR-15b-3p |  | 0.489921167 | 3.53E-06 |
| mmu-miR-140-5p |  | 0.48875018 | 0.022257 |
| mmu-let-7d-5p |  | 0.488468915 | 7E-125 |
| mmu-miR-34c-5p |  | 0.487314545 | 0 |
| mmu-miR-465c-5p |  | 0.486805928 | 7.2E-10 |
| mmu-miR-363-3p |  | 0.486731341 | 1.5E-125 |
| mmu-miR-148b-5p |  | 0.48647246 | 5.32E-05 |
| mmu-miR-290-5p |  | 0.4864354 | 0 |
| mmu-miR-466b-5p |  | 0.486271037 | 0.001568 |
| mmu-miR-466o-5p |  | 0.486271037 | 0.001568 |
| mmu-miR-145-5p |  | 0.484631868 | 8.17E-07 |
| mmu-miR-92a-1-5p |  | 0.483817319 | 5.02E-68 |
| mmu-miR-195-5p |  | 0.482997578 | 4.35E-15 |
| mmu-let-7f-5p |  | 0.4829649 | 0 |
| mmu-miR-322-5p |  | 0.480744877 | 6.55E-05 |
| mmu-miR-423-3p |  | 0.478842148 | 4.5E-66 |
| mmu-miR-181c-3p |  | 0.475398274 | 5.8E-34 |
| mmu-miR-25-5p |  | 0.474357065 | 0 |
| mmu-miR-101b-3p |  | 0.472550248 | 0 |
| mmu-miR-200b-3p |  | 0.471390216 | 4.13E-37 |
| mmu-miR-497-5p |  | 0.47015681 | 7.92E-21 |
| mmu-miR-196a-1-3p |  | 0.468409185 | 4.55E-11 |
| mmu-miR-410-3p |  | 0.467804485 | 1.04E-24 |
| mmu-miR-667-5p |  | 0.466177135 | 0.001334 |
| mmu-miR-1198-5p |  | 0.465210944 | 7.18E-05 |
| mmu-miR-25-3p |  | 0.465167817 | 0 |
| mmu-miR-466g |  | 0.463308524 | 0.004303 |
| mmu-miR-301b-5p |  | 0.457284996 | 0.005605 |
| mmu-miR-676-5p |  | 0.456467984 | 6.29E-06 |
| mmu-miR-700-5p |  | 0.455968688 | 0.000372 |
| mmu-miR-191-5p |  | 0.454639579 | 0 |
| mmu-miR-23a-3p |  | 0.452855138 | 1.9E-253 |
| mmu-miR-219-1-3p |  | 0.452769471 | 2.06E-12 |
| mmu-miR-351-5p |  | 0.450808383 | 0.002908 |
| mmu-miR-129-5p |  | 0.450796888 | 2.38E-09 |
| mmu-miR-294-5p |  | 0.450266943 | 0 |
| mmu-miR-130b-3p |  | 0.445821874 | 5E-257 |
| mmu-miR-7a-5p |  | 0.442570204 | 7.7E-152 |
| mmu-miR-182-3p |  | 0.441958852 | 2.52E-08 |
| mmu-miR-542-3p |  | 0.439374473 | 1.07E-06 |
| mmu-miR-146b-5p |  | 0.437872087 | 1.4E-113 |
| mmu-miR-28-3p |  | 0.437212723 | 8.01E-14 |
| mmu-miR-877-5p |  | 0.436596261 | 5.77E-92 |
| mmu-miR-148b-3p |  | 0.43616181 | 1.89E-83 |
| mmu-let-7c-5p |  | 0.434755671 | 0 |
| mmu-miR-199a-3p |  | 0.43377422 | 4.1E-100 |
| mmu-miR-199b-3p |  | 0.43377422 | 4.1E-100 |
| mmu-miR-210-3p |  | 0.433158001 | 1.9E-111 |
| mmu-miR-673-5p |  | 0.431460254 | 4.09E-06 |
| mmu-miR-30c-5p |  | 0.429661237 | 1.27E-35 |
| mmu-miR-205-5p |  | 0.429487986 | 2.28E-09 |
| mmu-miR-409-5p |  | 0.428094415 | 7.49E-19 |
| mmu-miR-192-5p |  | 0.426681765 | 0 |
| mmu-let-7g-5p |  | 0.423808868 | 0 |
| mmu-miR-677-5p |  | 0.422471595 | 4.15E-07 |
| mmu-let-7a-5p |  | 0.421999905 | 0 |
| mmu-miR-344-3p |  | 0.421458005 | 0.000255 |
| mmu-miR-425-5p |  | 0.420268217 | 4.85E-14 |
| mmu-miR-1224-5p |  | 0.41981399 | 1.13E-05 |
| mmu-miR-1947-5p |  | 0.419451276 | 7.15E-07 |
| mmu-miR-125a-3p |  | 0.415190633 | 0.000445 |
| mmu-miR-5117-3p |  | 0.415190633 | 0.000445 |
| mmu-miR-128-3p |  | 0.413712717 | 0 |
| mmu-miR-370-5p |  | 0.411308802 | 6.4E-05 |
| mmu-miR-34b-3p |  | 0.409909909 | 0.002917 |
| mmu-miR-149-3p |  | 0.409841264 | 2.03E-17 |
| mmu-miR-27b-3p |  | 0.409595085 | 2.1E-290 |
| mmu-miR-200a-3p |  | 0.408823579 | 8.86E-60 |
| mmu-miR-18a-3p |  | 0.407864537 | 0.00108 |
| mmu-miR-541-5p |  | 0.406344911 | 0 |
| mmu-miR-126-5p |  | 0.405963014 | 0.000292 |
| mmu-miR-129-2-3p |  | 0.405418177 | 9.33E-42 |
| mmu-miR-493-5p |  | 0.403245707 | 5.41E-10 |
| mmu-miR-760-3p |  | 0.402183776 | 2.61E-27 |
| mmu-miR-708-5p |  | 0.401650367 | 2E-293 |
| mmu-miR-345-5p |  | 0.400654621 | 5.97E-09 |
| mmu-miR-296-5p |  | 0.400017281 | 4.13E-92 |
| mmu-let-7i-5p |  | 0.397583737 | 7.68E-60 |
| mmu-miR-411-3p |  | 0.397131483 | 3.42E-24 |
| mmu-miR-3086-5p |  | 0.397118107 | 0.010154 |
| mmu-miR-127-5p |  | 0.395029804 | 4.33E-27 |
| mmu-miR-666-5p |  | 0.393904215 | 1.33E-26 |
| mmu-miR-744-5p |  | 0.391920877 | 0 |
| mmu-miR-143-3p |  | 0.391489584 | 4.2E-131 |
| mmu-miR-380-3p |  | 0.390184661 | 9.56E-49 |
| mmu-miR-27a-3p |  | 0.388685305 | 5E-254 |
| mmu-miR-369-3p |  | 0.387828446 | 3.8E-40 |
| mmu-miR-152-3p |  | 0.383491179 | 2.7E-106 |
| mmu-miR-3062-5p |  | 0.382935705 | 2.2E-05 |
| mmu-miR-335-3p |  | 0.381061978 | 6.14E-14 |
| mmu-miR-3064-5p |  | 0.380819439 | 1.11E-06 |
| mmu-miR-3079-5p |  | 0.37988185 | 0.006433 |
| mmu-miR-669f-3p |  | 0.378201096 | 0.009039 |
| mmu-miR-758-3p |  | 0.378201096 | 0.009039 |
| mmu-miR-674-5p |  | 0.378014021 | 3.4E-266 |
| mmu-miR-222-3p |  | 0.377914251 | 3.38E-82 |
| mmu-miR-221-5p |  | 0.375039311 | 0.000555 |
| mmu-miR-1193-5p |  | 0.374976433 | 2.23E-89 |
| mmu-miR-329-5p |  | 0.374158501 | 2.59E-28 |
| mmu-miR-674-3p |  | 0.371749125 | 4.14E-41 |
| mmu-miR-92a-3p |  | 0.371597917 | 0 |
| mmu-miR-431-5p |  | 0.36984486 | 3.17E-21 |
| mmu-let-7e-5p |  | 0.364725403 | 0 |
| mmu-miR-150-5p |  | 0.36133923 | 1.14E-19 |
| mmu-miR-465a-3p |  | 0.359794845 | 1.15E-10 |
| mmu-miR-465b-3p |  | 0.359794845 | 1.15E-10 |
| mmu-miR-465c-3p |  | 0.359794845 | 1.15E-10 |
| mmu-miR-470-5p |  | 0.359506595 | 6.27E-09 |
| mmu-miR-3057-5p |  | 0.357403446 | 3.64E-06 |
| mmu-miR-3068-5p |  | 0.357392207 | 0.0079 |
| mmu-miR-3102-5p.2-5p | | 0.355635042 | 3.44E-12 |
| mmu-miR-322-3p |  | 0.353904578 | 5.87E-72 |
| mmu-miR-423-5p |  | 0.352902308 | 0 |
| mmu-miR-193b-3p |  | 0.350945714 | 1.87E-08 |
| mmu-miR-503-5p |  | 0.350182142 | 9.3E-234 |
| mmu-miR-1195 |  | 0.349646692 | 1.49E-16 |
| mmu-miR-3095-3p |  | 0.349475165 | 2.7E-129 |
| mmu-miR-708-3p |  | 0.348698064 | 9.28E-42 |
| mmu-miR-376a-3p |  | 0.345967418 | 0 |
| mmu-miR-466b-3p |  | 0.34542746 | 1.77E-31 |
| mmu-miR-466c-3p |  | 0.34542746 | 1.77E-31 |
| mmu-miR-770-3p |  | 0.345235879 | 1.17E-32 |
| mmu-miR-139-3p |  | 0.345123498 | 1.69E-15 |
| mmu-miR-466e-3p |  | 0.344762695 | 8.28E-32 |
| mmu-miR-376b-3p |  | 0.344736139 | 5.68E-45 |
| mmu-miR-466p-3p |  | 0.344550993 | 4.35E-31 |
| mmu-miR-1193-3p |  | 0.342367257 | 2.69E-06 |
| mmu-miR-185-5p |  | 0.342294456 | 0 |
| mmu-miR-187-3p |  | 0.342114145 | 6.76E-35 |
| mmu-miR-449c-5p |  | 0.341535676 | 6.31E-10 |
| mmu-miR-369-5p |  | 0.340397409 | 3.03E-38 |
| mmu-miR-466a-3p |  | 0.339429912 | 2.3E-31 |
| mmu-miR-668-3p |  | 0.338207186 | 8.85E-15 |
| mmu-miR-881-3p |  | 0.336973926 | 0.000359 |
| mmu-miR-219-5p |  | 0.336042968 | 6.83E-06 |
| mmu-miR-433-5p |  | 0.335650671 | 9.98E-43 |
| mmu-miR-543-5p |  | 0.335346165 | 2.81E-05 |
| mmu-miR-412-5p |  | 0.334538369 | 3.99E-65 |
| mmu-miR-125a-5p |  | 0.333891831 | 1.62E-69 |
| mmu-miR-1930-5p |  | 0.321471897 | 3.22E-05 |
| mmu-miR-487b-3p |  | 0.321342327 | 5.1E-168 |
| mmu-miR-375-3p |  | 0.320759167 | 4.93E-11 |
| mmu-miR-3102-3p |  | 0.319345959 | 2.02E-19 |
| mmu-miR-431-3p |  | 0.318627146 | 4.17E-49 |
| mmu-miR-215-5p |  | 0.317712919 | 0.000412 |
| mmu-miR-434-3p |  | 0.316949485 | 0 |
| mmu-miR-411-5p |  | 0.314782301 | 6.1E-300 |
| mmu-miR-770-5p |  | 0.312900275 | 0.000182 |
| mmu-miR-664-5p |  | 0.312137922 | 6.62E-26 |
| mmu-miR-328-5p |  | 0.312036662 | 0.000563 |
| mmu-miR-434-5p |  | 0.311343257 | 2.06E-13 |
| mmu-miR-365-3p |  | 0.307341406 | 9.12E-24 |
| mmu-miR-23a-5p |  | 0.30656161 | 1.45E-12 |
| mmu-miR-337-3p |  | 0.305163528 | 4.63E-20 |
| mmu-miR-219-2-3p |  | 0.300058707 | 1.93E-19 |
| mmu-miR-351-3p |  | 0.299018736 | 5.66E-10 |
| mmu-miR-496-3p |  | 0.298436217 | 2.48E-35 |
| mmu-miR-126-3p |  | 0.297845573 | 0.001046 |
| mmu-miR-130b-5p |  | 0.296527865 | 1.16E-16 |
| mmu-miR-9-3p |  | 0.296380791 | 3.23E-08 |
| mmu-miR-5100 |  | 0.295740153 | 5.26E-11 |
| mmu-miR-127-3p |  | 0.295100244 | 0 |
| mmu-miR-376c-3p |  | 0.294358801 | 2.42E-33 |
| mmu-miR-9-5p |  | 0.293001875 | 4.87E-35 |
| mmu-miR-672-5p |  | 0.292157919 | 0 |
| mmu-miR-433-3p |  | 0.291553454 | 0 |
| mmu-miR-297a-3p |  | 0.290133657 | 2.2E-41 |
| mmu-miR-297b-3p |  | 0.290133657 | 2.2E-41 |
| mmu-miR-297c-3p |  | 0.290133657 | 2.2E-41 |
| mmu-miR-184-3p |  | 0.290012269 | 5.97E-89 |
| mmu-miR-337-5p |  | 0.287483751 | 1.1E-152 |
| mmu-miR-139-5p |  | 0.285929952 | 0.000616 |
| mmu-miR-665-3p |  | 0.28524116 | 6.1E-141 |
| mmu-miR-193b-5p |  | 0.283663567 | 2.24E-16 |
| mmu-miR-382-5p |  | 0.282501047 | 0 |
| mmu-miR-300-3p |  | 0.282490493 | 3.33E-22 |
| mmu-miR-466h-3p |  | 0.280891507 | 4.08E-19 |
| mmu-miR-379-5p |  | 0.278876513 | 0 |
| mmu-miR-541-3p |  | 0.278686355 | 1.33E-07 |
| mmu-miR-465a-5p |  | 0.27795075 | 0.001922 |
| mmu-miR-494-5p |  | 0.27795075 | 0.001922 |
| mmu-miR-92b-5p |  | 0.277096595 | 1E-207 |
| mmu-miR-299-3p |  | 0.277029136 | 2.32E-55 |
| mmu-miR-136-5p |  | 0.276867608 | 2.15E-51 |
| mmu-miR-1306-5p |  | 0.27608303 | 5.82E-22 |
| mmu-miR-1197-3p |  | 0.274944206 | 6.42E-13 |
| mmu-miR-449a-5p |  | 0.274555027 | 2.2E-10 |
| mmu-miR-187-5p |  | 0.274269967 | 8.1E-17 |
| mmu-miR-467a-3p |  | 0.274093348 | 3.5E-144 |
| mmu-miR-467d-3p |  | 0.274093348 | 3.5E-144 |
| mmu-miR-495-3p |  | 0.269103989 | 0 |
| mmu-miR-532-3p |  | 0.268196449 | 1.53E-27 |
| mmu-miR-380-5p |  | 0.267938342 | 7.64E-11 |
| mmu-miR-409-3p |  | 0.267164486 | 0 |
| mmu-miR-382-3p |  | 0.264003271 | 0 |
| mmu-miR-350-3p |  | 0.261169815 | 3.53E-61 |
| mmu-miR-377-5p |  | 0.259937831 | 1.75E-33 |
| mmu-miR-3072-3p |  | 0.259345314 | 1.16E-18 |
| mmu-miR-212-5p |  | 0.256965257 | 2.3E-05 |
| mmu-miR-465b-5p |  | 0.256965257 | 2.3E-05 |
| mmu-miR-154-5p |  | 0.256313294 | 4.12E-31 |
| mmu-miR-365-1-5p |  | 0.252734057 | 1.15E-06 |
| mmu-miR-103-2-5p |  | 0.252680332 | 0.000632 |
| mmu-miR-485-5p |  | 0.251170578 | 0 |
| mmu-miR-194-5p |  | 0.250814397 | 6.08E-13 |
| mmu-miR-323-3p |  | 0.250330708 | 0 |
| mmu-miR-7a-1-3p |  | 0.250048048 | 1.47E-14 |
| mmu-miR-342-3p |  | 0.248873466 | 0 |
| mmu-miR-543-3p |  | 0.2471372 | 0 |
| mmu-miR-323-5p |  | 0.244198244 | 8.91E-81 |
| mmu-miR-466m-3p |  | 0.243154774 | 1.68E-07 |
| mmu-miR-3072-5p |  | 0.24196451 | 2.41E-20 |
| mmu-miR-299-5p |  | 0.24173398 | 4.61E-33 |
| mmu-miR-1839-3p |  | 0.24171316 | 0.000358 |
| mmu-miR-34c-3p |  | 0.24171316 | 0.000358 |
| mmu-miR-136-3p |  | 0.240606309 | 6.19E-40 |
| mmu-miR-669l-3p |  | 0.238230022 | 0.000826 |
| mmu-miR-540-3p |  | 0.23520558 | 4.13E-87 |
| mmu-miR-467e-3p |  | 0.230899197 | 1.13E-12 |
| mmu-miR-134-5p |  | 0.226823584 | 0 |
| mmu-miR-1188-5p |  | 0.22660931 | 3.16E-48 |
| mmu-miR-1a-3p |  | 0.221729149 | 0 |
| mmu-miR-539-5p |  | 0.217186832 | 4.66E-19 |
| mmu-miR-129-1-3p |  | 0.216605672 | 1.59E-10 |
| mmu-miR-92b-3p |  | 0.215699985 | 0 |
| mmu-miR-154-3p |  | 0.21390783 | 1.4E-126 |
| mmu-miR-379-3p |  | 0.208024441 | 2.08E-13 |
| mmu-miR-494-3p |  | 0.206249119 | 0 |
| mmu-miR-130a-5p |  | 0.202981507 | 1.63E-14 |
| mmu-miR-466f-3p |  | 0.197557393 | 1.72E-88 |
| mmu-miR-1943-5p |  | 0.194516117 | 9.56E-09 |
| mmu-miR-381-3p |  | 0.192532022 | 2.47E-06 |
| mmu-miR-574-5p |  | 0.190594807 | 4.11E-05 |
| mmu-miR-370-3p |  | 0.189674839 | 6.51E-22 |
| mmu-miR-377-3p |  | 0.189400648 | 2.53E-21 |
| mmu-miR-880-3p |  | 0.189145475 | 0.00017 |
| mmu-miR-669h-3p |  | 0.187744458 | 7.13E-10 |
| mmu-miR-181b-5p |  | 0.187269051 | 2.7E-224 |
| mmu-miR-296-3p |  | 0.176737432 | 0 |
| mmu-miR-206-3p |  | 0.175014392 | 6.03E-11 |
| mmu-miR-467c-3p |  | 0.173723316 | 1.55E-06 |
| mmu-miR-181a-5p |  | 0.173117679 | 1.9E-161 |
| mmu-miR-410-5p |  | 0.162682308 | 1.56E-29 |
| mmu-miR-758-5p |  | 0.155736036 | 1.69E-54 |
| mmu-miR-466i-3p |  | 0.15427431 | 9.53E-26 |
| mmu-miR-485-3p |  | 0.147807399 | 0 |
| mmu-miR-669a-3p |  | 0.147505002 | 2.3E-238 |
| mmu-miR-669o-3p |  | 0.147505002 | 2.3E-238 |
| mmu-miR-669i |  | 0.147120492 | 3.62E-06 |
| mmu-miR-667-3p |  | 0.131280996 | 1.43E-24 |
| mmu-miR-495-5p |  | 0.125391072 | 8.7E-09 |
| mmu-miR-181a-2-3p |  | 0.122927618 | 5.04E-18 |
| mmu-miR-669m-3p |  | 0.104892117 | 1.25E-12 |
| mmu-miR-210-5p |  | 0.094676331 | 8.2E-35 |

**Supplementary Table 4. Real-time PCR primers. List of all primers used for detection of microRNA or gene expression levels by real-time PCR.**

| Gene name |  | Primer sequences (5’ to 3’) |
| --- | --- | --- |
| Nanog | Forward | CACCCACCCATGCTAGTCTT |
|  | Reverse | ACCCTCAAACTCCTGGTCCT |
| Oct4 | Forward | AGAGGGAACCTCCTCTGAGC |
|  | Reverse | TTCTAGCTCCTTCTGCAGGG |
| Sox2 | Forward | GAGTGGAAACTTTTGTCCGAGA |
|  | Reverse | GAAGCGTGTACTTATCCTTCTTCAT |
| Klf4 | Forward | GGCGAGTCTGACATGGCTG |
|  | Reverse | GCTGGACGCAGTGTCTTCTC |
| c-myc | Forward | ATGCCCCTCAACGTGAACTTC |
|  | Reverse | CGCAACATAGGATGGAGAGCA |
| Tfcp2l1 | Forward | CAGCCCGAACACTACAACCAG |
|  | Reverse | CAGCCGGATTTCATACGACTG |
| Prdm14 | Forward | TTGGTGATGTGCCACACTTT |
|  | Reverse | TCCAGTTCCCAGAACCTTTG |
| Tcl1 | Forward | GTGTACTTGGATGAGTTTCGTCG |
|  | Reverse | TTGCCACATTAAAGGCAGCTC |
| Wnt8a | Forward | GGGAACGGTGGAATTGTCCTG |
|  | Reverse | GCAGAGCGGATGGCATGAA |
| Dusp4 | Forward | CGTGCGCTGCAATACCATC |
|  | Reverse | CTCATAGCCACCTTTAAGCAGG |
| Egr1 | Forward | CCACAACAACAGGGAGACCT |
|  | Reverse | ACTGAGTGGCGAAGGCTTTA |
| Universal (miRNA) | Reverse | TGAATCGAGCACCAGTTACGCATGCCGAGGTCGACTTCCTAGA |
| U6 | Forward | CTCGCTTCGGCAGCACA |
| miR-296-3P | Forward | GAGGGTTGGGTGGAGGC |
| miR-222-3p | Forward | GGGAGCTACATCTGGCTA |
| miR-382-5p | Forward | GGGGAAGTTGTTCGTGGTGG |
| miR-92a-3p | Forward | GGTATTGCACTTGTCCC |
| miR-101b-3p | Forward | GGGGGTACAGTACTGTGATAG |
| miR-323-3p | Forward | GCACATTACACGGTCG |
| miR-411-5p | Forward | GGGGGTAGTAGACCGTATAG |
| miR-181d-3p | Forward | CCCACCGGGGGATGAA |
| miR-467a-5p | Forward | TAAGTGCCTGCATGTATATGC |
| miR-124-3p | Forward | GGTAAGGCACGCGGTGAAT |
| miR-331-3p | Forward | GCCCCTGGGCCTAT |
